# 

**Published:** 1896-03

**Authors:** 


### THE BRISTOL

### Medico-Chirurgical Journal.

A JOURNAL OF THE MEDICAL SCIENCES FOR THE WEST OF ENGLAND AND SOUTH WALES

*PUBLISHED UNDER THE AUSPICES OF*

*THE BRISTOL MEDICO-CHIRURGICAL SOCIETY.*

EDITOR:
R. SHINGLETON SMITH. M.D., B.Sc.

WITH WHOM ARE ASSOCIATED
J. MICHELL CLARKE, M.A., M.D.
F. R. CROSS, M.B. AND W. H. HARSANT.

ASSISTANT-EDITOR:
L. M. GRIFFITHS.

EDITORIAL SECRETARY:
B. M. H. ROGERS, B.A., M.D., B.Ch.

"Scire est nescire, nisi id me

Scire alius sciret."

*VOL. XIV.*

BRISTOL: J. W. ARROWSMITH.
London: J. & A. Churchill, 7 Great Marlborough Street.
1896.

R31
B8

J. W. Arrowsmith Publisher Bristol

### CONTENTS OF VOLUME XIV.

---

| | Page |
|---|---|
| Editorial Note ... ... ... ... ... ... ... ... ... ... ... ... ... ... | 1 |
| Induced Premature Labour in Certain Diseases of the Mother not Obstructing Delivery. By J. G. SWAYNE, M.D. Lond. ... 2, | 126 |
| The Use of Alcohol from a Medical Standpoint. By JOHN DACRE, M.R.C.S., Eng., L.R.C.P. Lond. ... ... ... ... ... ... ... | 10 |
| Two Years' Experience of the Operation for Strangulated Hernia at the Bristol General Hospital: and its Lessons. By CHARLES A. MORTON, F.R.C.S. Eng. ... ... ... ... ... ... ... ... ... ... | 23 |
| Extra-peritoneal Suppuration in Perforating Appendicitis. By J. LACY FIRTH, M.D., M.S. Lond., F.R.C.S. Eng. ... ... ... ... | 36 |
| Notes on a Case of Exophthalmic Goitre. By F. H. EDGEWORTH, B.A., M.B. Cantab., B.Sc. Lond. ... ... ... ... ... ... ... | 41 |
| The Clinical Significance of the Human Hand. By ARTHUR S. WOHLMANN, M.D., B.S. Lond. ... ... ... ... ... ... ... ... ... | 117 |
| Chloroform or Ether? By JOHN FREEMAN, F.R.C.S. Ed. ... ... | 137 |
| Dermoid Cyst of a Branchial Cleft. By THOMAS CARWARDINE, M.B., M.S. Lond., F.R.C.S. Eng. ... ... ... ... ... ... ... | 145 |
| A Case of Ligature and Division of the Vasa Deferentia for Prostatic Hypertrophy. By C. W. J. BRASHER, M.R.C.S. Eng., L.R.C.P. Lond. ... ... ... ... ... ... ... ... ... ... ... ... | 150 |
| The Causation and Treatment of Sudden Dyspnœa in Goitre. By CHARLES A. MORTON, F.R.C.S. Eng. ... ... ... ... ... ... ... | 213 |
| A Case of Hystero-Epilepsy. By BONVILLE B. FOX, M.A., M.D. Oxon. ... ... ... ... ... ... ... ... ... ... ... ... ... ... | 224 |
| On the Use of Oxygen in the Treatment of Anæmia, with a Case Treated by Transfusion of Blood. By J. MICHELL CLARKE, M.A., M.D. Cantab., F.R.C.P. Lond. ... ... ... ... ... ... | 232 |

Page

A Case of Needle in Foot Revealed by the X Rays. By W. E. POUNTNEY, M.D. ... ... ... ... ... ... ... ... ... ... ... 238

The Evils of Marriage and Pregnancy in Women who do not possess Sound Pelvic Organs. By A. E. AUST LAWRENCE, M.D., C.M. Aberd.... ... ... ... ... ... ... ... ... ... ... 289

A Case of Double Spasmodic Torticollis: Excision of both Spinal Accessory Nerves. By R. SHINGLETON SMITH, M.D., B.Sc. Lond., F.R.C.P. Lond. ... ... ... ... ... ... ... ... ... ... 297

The Diagnosis and Treatment of Lumbago. By F. H. EDGEWORTH, B.A., M.B. Cantab., B.Sc. Lond. ... ... ... ... ... ... ... 304

Notes on Self-Poisoning and its Treatment, with Special Reference to some Physical Methods. By C. J. WHITBY, B.A., M.D. Cantab. ... ... ... ... ... ... ... ... ... ... ... ... ... 307

PROGRESS OF THE MEDICAL SCIENCES ... ... ... 47, 155, 240, 317

REVIEWS OF BOOKS ... ... ... ... ... ... ... 71, 173, 256, 343

NOTES ON PREPARATIONS FOR THE SICK ... ... ... 87, 187, 277, 368

MEETINGS OF SOCIETIES ... ... ... ... ... ... ... 91, 191, 370

THE LIBRARY OF THE BRISTOL MEDICO-CHIRURGICAL SOCIETY ... ... ... ... ... ... ... ... ... 104, 206, 281, 373

LOCAL MEDICAL NOTES ... ... ... ... ... ... 110, 209, 284, 378

SCRAPS ... ... ... ... ... ... ... ... ... ... 113, 211, 287, 380

INTERNATIONAL CONGRESS OF DERMATOLOGY... ... ... ... ... ... 210

INTERNATIONAL CONGRESS OF GYNÆCOLOGY AND OBSTETRICS ... ... 210

JENNER ... ... ... ... ... ... ... ... ... ... ... ... ... ... 255

(*To be continued.*)

---

PELZER'S METHOD OF INDUCING LABOUR.

Dr. B. M. Hypes (*American Journal of Obstetrics*, December, 1895) says that Pelzer's method of inducing labour by intra-uterine injections of glycerine should be abandoned or the dosage reduced, especially in subjects with prior existing kidney affections.

A useful summary of many articles on Pelzer's method is given in the *Medical Chronicle* for February, 1896.

### Torquay Medical Society.

*The following donations have been received since the publication of the list in December:*

*February 29th, 1896.*

| | |
|---|---|
| Bootle Public Library Committee (1) ... ... ... | 75 volumes. |
| F. R. Cross (2) ... ... ... ... ... ... ... ... ... | 20 ,, |
| George M. Gould, M.D. (3) ... ... ... ... ... ... | 5 ,, |
| L. M. Griffiths (4) ... ... ... ... ... ... ... ... | 11 ,, |
| Council of the Ophthalmological Society (5) ... | 13 ,, |
| Mrs. R. B. Ruddock (6) ... ... ... .. ... ... | 1 volume. |
| E. J. Sheppard (7) ... ... ... ... ... ... ... | 53 volumes. |
| J. Greig Smith (8) ... ... ... ... ... ... ... | 1 volume. |
| R. Shingleton Smith, M.D. (9) ... ... ... ... | 5 volumes. |
| The Surgeon-General, United States Army (10)... | 1 volume. |
| P. Watson Williams, M.D. (11) ... ... ... ... | 1 ,, |

---

### NINETEENTH LIST OF BOOKS.

The titles of books mentioned in previous lists are not repeated.

The figures in brackets refer to the figures after the names of the donors, and show by whom the volumes were presented. The books to which no such figures are attached have either been bought from the Library Fund or received through the *Journal*.

*Anatomical Examinations.* Vol. II. ... ... ... ... ... ... (7) 6th Ed. 1824
**Arnold, T. K.** ... *Practical Introduction to Greek Accidence* (7) 5th Ed. 1852
**Arnott, N.** ... ... *Elements of Physics* ... ... ... ... ... ... (7) 1827
**Ashhurst, J.** [Ed.] *The International Encyclopædia of Surgery* Vol. VII. 1895
**Averill, C.** ... ... *Operative Surgery* ... ... ... ... ... ... ... ... (1) 1823
**Babington and J. Curry, W.** *Outlines of a Course of Lectures on the Practice of Medicine* ... ... ... ... ... ... ... (7) 1802-6
**Barry, E.** ... ... *On Digestions and Discharges of the Human Body* (1) 1759
**Bateman, T.** ... *Cutaneous Diseases* ... ... ... ... ... ... (7) 3rd Ed. 1814
**Beale, V. D. Harris and E. C.** *Pulmonary Consumption* ... ... ... ... 1895
**Bell, B.** ... ... *On Ulcers* ... ... ... ... ... ... ... (1) 4th Ed. 1787
**Bell, J.** ... ... *The Anatomy of the Human Body.* Vol. II. (7) 2nd Ed. 1802
**Bichat, X.** ... ... *General Anatomy* (Tr. by C. Coffyn, Ed. by G. Calvert). 2 vols. ... ... ... ... ... ... ... (1) 1824
**Blundell, J.** ... ... *Lectures on Midwifery, and the Diseases of Women and Children*... ... ... ... ... .. ... ... (7) 1832
**Boerhaave, [H]** ... *Aphorisms* (Translated) ... ... ... ... ... ... (1) 1735
*Botanick Garden at Chelsea, Memoirs of the* ... ... ... ... ... ... (7) 1820
**Brande, W. T.** ... *A Manual of Pharmacy* ... ... ... ... (7) 3rd Ed. 1833
**Bridge, Rev. B.** ... *Elements of Algebra* ... ... ... ... (7) 7th Ed. 1831
**Brisseau-Mirbel, C. F.** *Élémens de Physiologie végétale et de botanique.* 3 vols. (7) 1815
**Brittan, [F.]** ... *Blood Diseases and Blood Germs* ... ... ... ... (6) 1874
**Brown, A. M.** ... *The Natural Arsenical Waters of La Bourboule* ... ... [N.D.]
**Burnes, A.** ... ... *Reise nach und in Bokhara.* Band I. ... ... (7) 1835
**Carlisle, A.** ... ... *On the Disorders of Old Age* ... ... ... ... ... ... (1) 1817
**Carpenter, E.** ... *Modern Science* ... ... ... ... ... ... ... ... (4) 1885
*Catalogue (Index) of Library of Surgeon-General's Office,* U.S. Army. Vol. XVI. Abbreviations of Titles of Medical Periodicals, etc. Vols. I.—XVI. ... ... (10) 1895
**Caton, T. M.** ... *On Nervous, Hypochondriac, and Hysterical Diseases* (7) [N.D.]
**Chapman, H. C.** ... *Manual of Medical Jurisprudence and Toxicology.* 2nd Ed. 1896
**Clement, W. J.** ... *Observations in Surgery and Pathology* ... ... ... (1) [1832]
**Cleveland, W. F.** *The Prophylactic Clothing of the Body* ... ... ... 1896
**Cooper, Sir A.** ... *Outlines of the Lectures on Surgery delivered at St. Thomas's and Guy's Hospitals* ... ... ... (1) 1822
**Cooper, S.** ... ... *Modern Surgery* ... ... ... ... ... ... ... ... (1) 1813
,, ... ... *A Dictionary of Practical Surgery* ... ... (1) 3rd Ed. 1818
**Crombie, J. L.** ... *Invalid's Recipe Book* ... ... ... ... ... ... (4) 1884
**Cullen, W.** ... ... *Synopsis Nosologiæ Methodicæ.* Tom. II. (7) 4th Ed. 1785
,, ... ... ,, ,, ... (1) Editio altera 1816
,, ... ... *Nosology* (Translated) ... .. ... ... (1) New Ed. 1815
,, ... ... *First Lines of the Practice of Physic.* 4 vols. (1) New Ed. 1786
**Cullingworth, C. J.** *Clinical Illustrations of the Diseases of the Fallopian Tubes and of Tubal Gestation* ... ... ... ... 1895
**Curry, W. Babington and J.** *Outlines of a Course of Lectures on the Practice of Medicine* ... ... ... ... ... ... ...(7) 1802-6
**Davies, H.** ... ... *Diseases of the Lungs and Heart* ... ... (9) 2nd Ed. 1854
**Denman, T.** ... ... *Aphorisms on the Application and Use of the Forceps and Vectis* ... ... ... ... ... (1) 6th Ed. 1817

**Dionis, [P.]** ... ... *A Course of Chirurgical Operations* (Translated) (1) 2nd Ed. 1733

*Dispensatory, The Edinburgh New* ... ... ... ... ... ... ... (1) 6th Ed. 1801

*Dissector, The London* ... ... ... ... (7) 4th Ed., 1813; (1) 5th Ed. 1816

**Doléris, J.-A.** ... *De l'Endométrite et de son Traitement* ... ... ... ... (4) 1887

**Dowse, T. S.** ... *Syphilis of the Brain and Spinal Cord* (2) 2nd Ed. 1881

**Dupouy, E.** ... ... *Prostitution in Antiquity* (Tr. by T. C. Minor) ... 1895

**Dutt, A. C.** ... ... *Health Notes for the Seaside* ... ... ... ... ... ... ... 1895

**Elliotson, J.** ... *The Introductory Lecture of a Course upon State-Medicine* ... ... ... ... ... ... ... ... (1) 1821

**Fonblanque, J. A. Paris and J. S. M.** *Medical Jurisprudence.* 3 vols. (7) 1823

**Forsyth, J. S.** ... *The New London Medical and Surgical Dictionary* (1) 1826

,, ... *A Dictionary of Diet* ... ... ... ... (1) 2nd Ed. 1834

**Fournier, A.** ... *Syphilis et Mariage* ... ... ... ... ... ... ... (2) 1880

**Freind, J.** ... ... *The History of Physick.* 2 vols. ... ... ... (1) 1725-26

**Fyfe, A.** ... ... *A Compendium of Anatomy.* 3 vols. ... (1) 6th Ed. 1815

**Gärtner, A.** ... ... *Leitfaden der Hygiene.* ... ... ... Zweite Auflage 1895

**Geoghegan, E.** ... *On Ruptures* ... ... ... ... ... ... ... ... ... ... (1) 1810

**Gibson, T.** ... ... *The Anatomy of Humane Bodies Epitomized* (1) 7th Ed. 1716

**Goldsmith, O.** ... *History of Greece.* Vol. I. ... ... ... (7) New Ed. 1796

,, ... *History of Rome.* Vol. I. ... ... ... (7) 11th Ed. 1812

**Good, J. M.** ... ... *The Study of Medicine.* 4 vols. ... ... (1) 4th Ed. 1840

**Graham, S.** ... ... *Lectures on the Science of Human Life*... ... ... (1) 1849

**Greene, R.** ... ... *The Schott Treatment for Chronic Heart Diseases* ... 1895

**Gregory, G.** ... ... *Theory and Practice of Medicine* ... ... (9) 5th Ed. 1839

**Guthrie, G. J.** ... *Lectures on the Operative Surgery of the Eye* (7) 2nd Ed. 1827

**Hahnemann, S.** ... *The Homœopathic Medical Doctrine* (Tr. by C. H. Devrient) ... ... ... ... ... ... ... ... (1) 1833

**Hall, M.** ... ... *Lectures on the Nervous System* ... ... ... ... (2) 1836

**Haller, A. v.** ... *Primæ Lineæ Physiologiæ* ... ... (1) Editio tertia 1765

**Hamilton, J.** ... *On Purgative Medicines* ... ... ... ... (1) 8th Ed. 1826

**Handley, J.** ... ... *Colloquia Chirurgica; or, The Art of Surgery Epitomiz'd* (7) 4th Ed. 1733

**Harris and E. C. Beale, V. D.** *Pulmonary Consumption* ... ... ... ... 1895

**Havers, C.** ... ... *Osteologia Nova, or some new Observations on the Bones.* (1) 1691

**Heister, L.** ... ... *A Compendium of Anatomy* (Translated) ... ... (1) 1752

*Hospital Pupil's Guide, The* ... ... ... ... ... ... ... ... (1) 2nd Ed. 1818

**Howship, J.** ... *Diseases of the Lower Intestines, and Anus* (7) 2nd Ed. 1821

,, ... *On the Diseases of the Urine and Urinary Organs* (7) 1823

**Hyde and F. H. Montgomery, J. N.** *Manual of Syphilis and the Venereal Diseases* ... ... ... ... ... ... ... ... ... ... 1895

**Impey, S. P.** ... *A Handbook on Leprosy* ... ... ... ... ... ... ... 1896

**Innes, J.** ... ... *A Short Description of the Human Muscles* (1) 8th Ed. 1815

**Jennings, J. E.** ... *Color-Vision and Color-Blindness* ... ... ... ... ... 1896

[**Jewel, G.**] ... ... *The London Practice of Midwifery*... ... (7) 4th Ed. 1816

**Johnson, Sir G.** ... *History of the Cholera Controversy* ... ... ... 1896

**Kidd, J.** ... ... *Physical Condition of Man* ... ... ... (2) [4th Ed.] 1836

**Kiernan, F.** ... ... *A Practical Treatise on the Venereal Disease* ... (7) 1815

**Laennec, R. T. H.** *Diseases of the Chest* (Tr. by J. Forbes) (7) 3rd Ed. 1829

**Langerhans, R.** ... *Grundriss der Pathologischen Anatomie.* Zweite Auflage 1896

| | | | |
|---|---|---|---|
| **Lawrence, W.** | *Lectures on Man* | (2) 7th Ed. | 1838 |
| *Leprosy Fund Prize Essays on Subjects connected with Leprosy, The National* | | Nos. 2, 3, 4 | 1895 |
| **Lersch, B. M.** | *Geschichte der Volksseuchen* | | 1896 |
| **Lind, J.** | *On Diseases incidental to Europeans in Hot Climates* | (1) 6th Ed. | 1808 |
| **Lobb, T.** | *A Treatise of the Small Pox* | (1) | 1731 |
| **Lockwood, C. B.** | *Aseptic Surgery* | | 1896 |
| ,, | *Traumatic Infection* | | 1896 |
| **Lockwood, G. R.** | *Manual of the Practice of Medicine* | | 1896 |
| **Mackenzie, P.** | *On Mineral Waters* | (1) 2nd Ed. | 1820 |
| **Male, G. E.** | *Forensic Medicine* | (1) 2nd Ed. | 1818 |
| **Marten, B.** | *A New Theory of Consumptions* | (1) | 1720 |
| **Monro, A.** | *The Anatomy of the Human Bones and Nerves* | (7) 5th Ed. | 1750 |
| **Monro, T. K.** | *History of Chronic Nervous Diseases* | | 1895 |
| **Montgomery, J. N. Hyde and F. H.** | *Manual of Syphilis and the Venereal Diseases* | | 1895 |
| **Morgan, T.** | *Philosophical Principles of Medicine* | (1) 2nd Ed. | 1730 |
| **Morris, R. T.** | *Lectures on Appendicitis. Notes* | | 1895 |
| **Mouat and H. S. Snell, F. J.** | *Hospital Construction and Management* | (2) | 1883 |
| **Mundé, P. F.** | *De l'Électricité comme Agent thérapeutique en Gynécologie* (Traduit et annoté par P. Ménière) | (4) | 1888 |
| **Munk, W.** | *The Life of Sir Henry Halford, Bart.* | | 1895 |
| **Murray, A.** | *A Description of the Arteries of the Human Body* (Tr. by A. Scott) | (7) | 1801 |
| **Neale, R.** | *Appendix to Medical Digest*, 1891-95 | | 1895 |
| **Noehden, G. H.** | *A Grammar of the German Language* | (7) 7th Ed. | 1835 |
| **Orfila, P.** | *On Poison* (Tr. by R. H. Black) | (1) 2nd Ed. | 1820 |
| **Paris and J. S. M. Fonblanque, J. A.** | *Medical Jurisprudence.* 3 vols. | (7) | 1823 |
| *Pasteur, Velada Fúnebre en honra del eminente sabio Francés Luis* | | | 1895 |
| *Pathology, An Atlas of Illustrations of* (New Syd. Soc.). Fasc. X. | | | 1895 |
| **Pavy, F. W.** | *The Physiology of the Carbohydrates.* An Epicriticism | | 1895 |
| *Pharmacopœia, The New Medico-Chirurgical* | | (1) 3rd Ed. | 1824 |
| **Phillips, R.** | *A Translation of the Pharmacopœia of the Royal College of Physicians of London*, 1824 | (1) (7) | 1824 |
| **Pole, T.** | *The Anatomical Instructor* | (1) New Ed. | 1813 |
| **Pott, P.** | *On Injuries to the Head* | (1) | 1768 |
| ,, | *On Fractures and Dislocations* | (1) | 1768 |
| *Public Health and the Removal of Nuisances, Practical Information as to the Laws relating to* | | (4) | 1876 |
| **Purdy, C. W.** | *Practical Uranalysis and Urinary Diagnosis* | 2nd Ed. | 1895 |
| **Quain, [J.]** | *Elements of Anatomy.* 10th Ed. (Ed. by E. A. Schäfer and G. D. Thane) | Vol. III., Part IV. | 1896 |
| **Quincy, J.** | *Lexicon Physico-Medicum* | (1) 3rd Ed. | 1726 |
| **Reid, J.** | *A Treatise on Consumption* | (7) | 1806 |
| **Rennie, J.** | *A New Supplement to the Pharmacopœias* | (1) (7) | 1826 |
| **Renwick, T.** | *The Continuation of the Narrative of Miss Margaret M'Avoy's Case* | (1) | 1820 |
| **Richerand, A.** | *Elements of Physiology* (Tr. by G. J. M. De Lys) | (7) 4th Ed. | 1824 |
| **Roberton, J.** | *On the Generative System* | (1) 4th Ed. | 1817 |
| **Roget, P. M.** | *Animal and Vegetable Physiology.* 2 vols. | (2) | 1834 |

| | | | |
|---|---|---|---|
| **Sanctorius, [S.]** ... | *Medicina Statica* (Tr. by J. Quincy) ... | (1) 5th Ed. | 1737 |
| **Sandwith, F. M.**... | *Egypt as a Winter Resort* ... ... ... ... ... | (11) | 1889 |
| **Senn, N.** ... ... | *Principles of Surgery* ... ... ... ... ... | 2nd Ed. | 1895 |
| **Shoemaker, J. V.** | *Materia Medica and Therapeutics* ... ... ... | 3rd Ed. | 1895 |
| **[Simmons, S. F.]**... | *Elements of Anatomy* ... ... ... ... | (1) 2nd Ed. | 1781 |
| **Smith, Sir J. E.** ... | *A Grammar of Botany* ... ... ... ... ... ... | (7) | 1821 |
| **Smith, W.** ... ... | *Nature Studied with a View to Preserve and Restore Health* ... ... ... ... ... ... ... ... | (7) | 1774 |
| **Snell, F. J. Mouat and H. S.** | *Hospital Construction and Management* | (2) | 1883 |
| **Starkweather, G. B.** | *The Law of Sex* .. ... ... ... ... ... ... | (2) | 1883 |
| **Startin, J.** [Ed.]... | *Skin Pharmacopœia* ... ... ... ... ... | 4th Ed. | 1896 |
| **Stokes, J.** ... ... | *A Botanical Materia Medica.* 4 vols. ... ... | (1) | 1812 |
| **Stone, A. D.** ... ... | *Diseases of the Stomach, and of Digestion* ... ... | (1) | 1806 |
| *Surgery, An American Text-Book of* (Ed. by W. W. Keen and J. W. White) | | 2nd Ed. | 1895 |
| **Syme, J.** ... ... | *Principles of Surgery* ... ... ... ... | (2) 3rd Ed. | 1842 |
| *Testament, The Greek, with English Notes.* By the Rev. S. T. Bloomfield. 2 vols. ... ... ... ... ... ... ... ... ... | | (7) | 1832 |
| **Thomas, R.** ... ... | *Practice of Physic* ... ... ... ... ... | (1) 7th Ed. | 1821 |
| **Thomson, A. T.** ... | *A Conspectus of the Pharmacopœias* ... | (1) 2nd Ed. | 1816 |
| **Thomson, J.** ... | *Lectures on Inflammation* ... ... ... .. ... | (1) | 1817 |
| ,, ... ... | *Life of Cullen.* 2 vols. ... ... ... ... ... | (2) | 1859 |
| **Travers, B.** ... ... | *Diseases of the Eye*... ... ... ... ... | (7) 3rd Ed. | 1824 |
| **Trotter, T.** ... ... | *A View of the Nervous Temperament* ... | (1) 3rd Ed. | 1812 |
| **Turnbull, A.** ... | *On Diseases of the Nerves, Eye and Ear* | (7) 3rd Ed. | 1837 |
| **Turnbull, J.**... ... | *On Disorders of the Stomach* ... ... ... ... ... | (1) | 1856 |
| **Vacher, F.** ... ... | *The Prevention of Epidemics* ... ... ... ... ... ... | | 1895 |
| **Willich, A. F. M.** | *Lectures on Diet and Regimen* ... ... | (1) 4th Ed. | 1809 |
| **Witkowski, G.-J.** | *Les Accouchements dans les Beaux-Arts, dans la Littérature et au Théatre* ... ... ... ... ... ... ... | | 1894 |

### TRANSACTIONS, REPORTS, JOURNALS, &c.

| | | |
|---|---|---|
| Advertiser's A B C, The ... ... ... ... ... ... ... ... ... ... ... ... | | 1896 |
| American Gynæcological and Obstetrical Journal, The ... | Vol. VII. | 1895 |
| American Journal of Obstetrics, The ... ... ... ... | Vol. XXXII. | 1895 |
| American Journal of the Medical Sciences, The ... ... | Vol. CX. | 1895 |
| American Practitioner and News, The ... ... ... ... | Vols. XIX., XX. | 1895 |
| American Year-Book of Medicine and Surgery, The ... ... ... ... | | 1896 |
| Annali di Ostetricia e Ginecologia... ... ... ... ... ... ... ... ... | | 1895 |
| Annual of the Universal Medical Sciences. 5 Vols. ... ... ... ... | | 1895 |
| Archives cliniques de Bordeaux ... ... ... ... ... ... | Tome IV. | 1895 |
| Archives de Neurologie ... ... ... ... ... ... ... ... ... | Tome XXX. | 1895 |
| Archives of Surgery ... ... ... ... ... ... ... ... ... ... ... | Vol. VI. | 1895 |
| Association of American Physicians, Transactions of the ... | Vol. X. | 1895 |
| Australian Medical Journal, The ... ... ... ... ... ... | Vol. XVII. | 1895 |
| Birmingham Medical Review, The ... ... ... ... | Vol. XXXVIII. | 1895 |
| Boston Medical and Surgical Journal, The ... ... ... | Vol. CXXXIII. | 1895 |
| Brain ... ... ... ... ... ... ... ... ... ... ... ... ... | (4) Vol. I. | 1879 |
| Bristol Health Report, 1887 ... ... ... ... ... ... ... ... ... | (4) | 1888 |

British Journal of Dermatology, The ... ... ... ... ... Vol. VII. 1895
British Medical Journal, The ... ... ... ... ... ... ... Vol. II. for 1895
Buffalo Medical and Surgical Journal ... ... .. ... (8) Vol. XXVIII. 1888
Centralblatt für Chirurgie ... ... .. ... ... ... ... ... ... ... 1895
Centralblatt für Gynäkologie ... ... ... ... ... ... ... ... ... 1895
Centralblatt für innere Medicin ... ... ... ... ... ... ... ... 1895
Cincinnati Lancet-Clinic, The ... ... ... ... ... Vol. LXXIV. 1895
Clinical Sketches ... ... ... ... ... ... ... ... ... ... Vol. II. 1895
Clinical Society of London, Transactions of the ... Vol. XXVIII. 1895
College of Physicians of Philadelphia, Transactions of the 3rd Ser. Vol. XVI. 1894
Colorado State Medical Society, Transactions of the ... ... ... ... 1895
Dermatological Society of Great Britain and Ireland, Transactions of the ... ... ... ... ... ... ... ... ... ... ... ... Vol. I. 1895
Dublin Journal of Medical Science, The ... ... ... ... ... Vol. C. 1895
France médicale, La ... ... ... ... ... ... ... ... ... ... ... ... 1895
Gazette des Hôpitaux ... ... ... ... ... ... ... ... ... ... ... 1895
Hazell's Annual ... ... ... ... ... ... ... ... ... ... ... ... 1896
Hospitals—

Johns Hopkins Hospital Reports, The ... Vols. IV., No. 9; V. 1895
Middlesex Hospital. Registrars' Reports for 1894 ... ... ... 1895

Iowa State Medical Society, Transactions of the ... ... Vol. XIII. 1895
Journal of Cutaneous and Genito-Urinary Diseases ... ... Vol. XIII. [1895]
Journal of Laryngology, Rhinology, and Otology, The ... ... Vol. IX. 1895
Journal of Nervous and Mental Disease, The ... ... ... Vol. XX. 1895
Journal of the American Medical Association, The ... ... Vol. XXV. 1895
Lancet, The... ... ... ... ... ... ... ... (2) 1879-80; Vol. II. for 1895
Library, The ... ... ... ... ... ... ... ... ... ... ... Vol. VII. 1895
Liverpool Medico-Chirurgical Journal, The ... ... ... Vol. XV. 1895
London and Provincial Medical Directory, The .. ... ... ... ... (9) 1868
Mathews' Medical Quarterly ... ... ... ... ... ... ... ... Vol. II. 1895
Medical Adviser, The ... .. ... ... ... ... (1) Vols. I.—III. 1824-25
Medical Age, The ... .. ... ... ... ... ... ... ... ... Vol. XIII. 1895
Medical and Surgical Reporter, The ... ... Vols. LXXII., LXXIII. 1895
Medical Association of the State of Alabama, Transactions of the ... 1895
Medical Directory, The ... ... ... ... ... ... ... ... ... ... ... 1896
Medical Magazine, The ... ... ... ... ... ... ... ... ... ... Vol. IV. 1895
Medical Press and Circular, The ... ... ... ... ... ... [Vol. CXI.] 1895
Medical Record, The (3) Vols. XIX.—XXIII., 1881-83; XLVIII. 1895
Medical Society of London, Transactions of the ... ... Vol. XVIII. 1895
Medical Society of New Jersey, Transactions of the ... ... ... ... 1895
Medical Society of the State of New York, Transactions of the ... ... 1895
Michigan State Medical Society, Transactions of the ... Vol. XIX. 1895
Münchener medicinische Wochenschrift ... ... ... ... ... ... ... 1895
New Hampshire Medical Society, Transactions of the ... ... ... ... 1895
New York Academy of Medicine, Constitution and By-laws, List of Fellows, &c. of the ... ... ... ... ... ... ... ... ... ... ... (4) 1891
New York Medical Journal, The ... ... ... ... ... ... ... Vol. LXII. 1895
Nord médical, Le ... ... ... ... ... ... ... ... ... ... ... Tome I. 1895
Occidental Medical Times ... ... ... ... ... ... ... ... ... Vol. IX. 1895

Ohio State Medical Society, Transactions of the Semi-Centennial Meeting of the ... 1895
Ophthalmological Transactions ... (5) Vols. I.—XIII. 1881–93
Post-Graduate, The ... Vol. X. [1895]
Practitioner, The ... Vol. LV. 1895
Progrès médical, Le ... 3e Sér. Tome II. 1895
Retrospect of Medicine, Braithwaite's ... Vol. CXII. 1896
Revue de Laryngologie, d'Otologie et de Rhinologie ... Tome XV. 1895
Revue de Thérapeutique médico-chirurgicale ... 1895
Revue générale d'Ophtalmologie ... Tome XIV. 1895
Revue internationale de Médecine et de Chirurgie pratiques ... 1895
Revue médicale, La ... 1894; 1895
St. Louis Medical and Surgical Journal, The ... Vol. LXIX. 1895
St. Petersburger medicinische Wochenschrift ... 1895
Teratologia ... Vol. II. 1895
Texas State Medical Association, Transactions of the ... 1895
Therapeutic Gazette, The ... Vol. XIX. 1895
Whitaker's Almanack ... 1896
Wiener klinische Wochenschrift ... 1895
Year-Book of Treatment, The ... 1896
Zeitschrift für Krankenpflege ... 1895

### Local Medical Notes.

BATH ROYAL UNITED HOSPITAL.

G. A. Bannatyne, M.D. Glasg., has been appointed Physician in the place of Dr. Biddlecombe resigned.

G. E. Bowker, M.B. Edin., has been appointed Honorary Assistant Medical Officer.

UNIVERSITY COLLEGE, BRISTOL.

The following appointments have been made:—*Professor of Pathology and Morbid Anatomy*—J. Michell Clarke, M.A., M.D. Cantab, M.R.C.P. *Lecturer in Practical Midwifery*—Walter C. Swayne, M.D. Lond. *Lecturer in Practical Physiology and Histology*—F. H. Edgeworth, B.A., M.B., B.C. Cantab, B.Sc. Lond.

The following students of University College have passed examinations:

**University of London.**—Intermediate Examination. *Physiology only*—First Class, H. T. S. Aveline. Second Class, N. S. V. Stock. Honours, J. H. Bodman (Third Class in Medicine, Second Class in Obstetric Medicine).

**Examining Board in England by the Royal Colleges of Physicians and Surgeons.**—First Examination, January, 1896. Part I. *Chemistry and Physics*—H. Hemsted, J. C. Norton, L. A. Moore, C. F. Walters. Part II. *Practical Pharmacy*—K. H. Beverley. Part III. *Elementary Biology*—J. C. Clayton. Second Examination. *Anatomy and Physiology*—W. T. Lord. *Physiology*—E. G. Bunbury. Third Examination. *Surgery*—C. A. Dykes, W. R. Gwynn,* J. C. Macwatters, G. E. F. Stammers.* *Midwifery*—E. R. Woodford.

* Completes Examination.

**Society of Apothecaries.**—Pass List. December, 1895. *Medicine, Forensic Medicine, and Midwifery*—A. T. Morgan. *Medicine*—J. H. R. Pigeon. *Forensic Medicine and Midwifery*—E. R. Bowen.

**Indian Medical Service.**—A. E. H. Pinch.

BRISTOL GENERAL HOSPITAL.

W. J. Penny, F.R.C.S. Eng., has been appointed Consulting Surgeon.

R. G. Poole Lansdown, M.D., B.S. Durh., has been appointed Surgeon.

J. Lacy Firth, M.D., M.S. Lond., F.R.C.S. Eng., has been appointed Assistant-Surgeon.

BRISTOL HOSPITAL FOR SICK CHILDREN AND WOMEN.

John Dacre, M.R.C.S. Eng., has been appointed Consulting Surgeon.

The following gentlemen have been appointed to hold office for one year:—

Assistant-Physicians—T. Fisher, M.D. Lond.; C. E. Williams, M.D. Cantab.

Assistant-Surgeons—C. A. Morton, F.R.C.S. Eng.; W. H. Kendall, M.R.C.S. Eng.

Registrar—John S. Griffiths, M.R.C.S. Eng.

CLIFTON SPA.

A very decided attempt is being made by Sir George Newnes to revive the faded, if not extinguished, glories of the old Hotwells spring. When the British Medical Association met here in 1894, the Pump-room was opened; but owing to numerous difficulties arising, all work ceased, both in building the baths and converting the existing houses into the Hydropathic Establishment. A company to run the concern has now been started, and we hear that no difficulty has been experienced in obtaining the necessary capital. When completed the whole place will equal, if not excel, any similar "Hydro" in the United Kingdom; for the baths—of which we have seen the plans—will be of the most recent and approved pattern.

We have been so accustomed to look upon the pump situated in a cave at the base of St. Vincent's rocks as the original well, that it came as a surprise to us to hear that Sir George had secured the real original well and was supplying its water at the Pump-room, free from contamination of the not too clean tidal waters of the Avon.

At the end of the last century the Hotwells was second only to Bath in popularity. From various accounts that we have seen two causes appear to have led to its decadence. Firstly, the inordinate rent imposed by the Merchant Venturers, the lords of the manor; and, secondly, the building of Clifton. All the big crescents were built during the last ten years of the eighteenth century, and Granby Hill—described by one physician as a dangerous and precipitous tract—was the only communication between the Hotwells and Clifton, and too exhausting for the invalids to descend and ascend three times a day. In a very short time the place was deserted; the Hotwell house fell out of repair, and was pulled down in 1822 when the Bridge Valley Road was made. An attempt was however made soon after to recall the visitors. A new and imposing house was built in "the Tuscan style"; but the opportunity had been lost, and the house continued its somewhat precarious existence till the year 1867, when it in turn was pulled down in order that the navigation of the river might be improved by removal of the Hotwells point. There are numerous interesting facts in connection with the popularity of the well, that we could, if space permitted, recount, and many contemporary writers and novelists (Fanny Burney, Smollett, &c.) describe, no doubt from personal experience, the gaieties and follies of the times. One peculiar phenomenon alone we will notice. On November 1st, 1755, the water of the well became turbid and red, while the ebbing tide flowed back. No one could account for this remarkable fact, till some time after when the news of the disastrous earthquake at Lisbon was reported.

### THE NEW "PHOTOGRAPHY."

On February 10th Mrs. L. consulted Mr. T. F. Edgeworth on account of pain in the front of the right wrist, due, she thought, to a needle which had accidentally penetrated the skin. There was some tenderness on pressure, but nothing further could be made out. Before any exploratory incision was made, Professor Wertheimer, the Principal of the Bristol Merchant Venturers' College, very kindly made a radiogram by Röntgen's rays. It showed the needle quite distinctly.

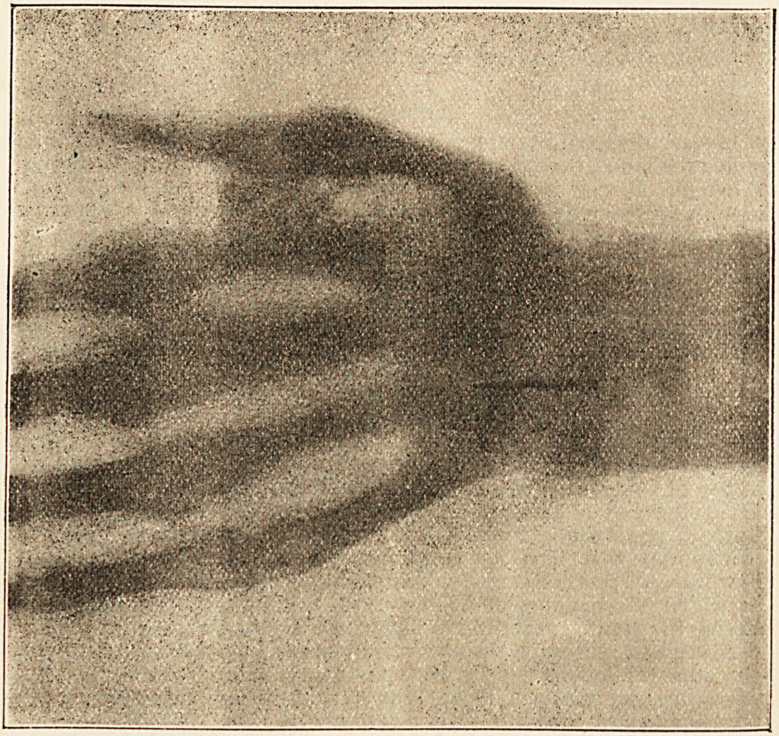

A few days subsequently, after an injection of cocaine, the needle was easily removed. The radiogram was of great assistance in showing that the needle was there, and also its exact position, so that an incision could be made just over the upper end of it.

This is the first case in Bristol in which surgical use has been made of the discovery.

### Bristol Medico-Chirurgical Society.

### President.

ARTHUR W. PRICHARD.

### President-Elect.

A. E. AUST LAWRENCE, M.D., C.M. Aberd.

### Honorary Secretary.

J. PAUL BUSH.

### Committee.

*Past Presidents.*

R. W. COE.
AUGUSTIN PRICHARD, M.D. Berlin.
J. G. SWAYNE, M.D. Lond.
E. LONG FOX, M.D. Oxon.
W. JOHNSTONE FYFFE, M.D., B.A. Dub.
W. H. SPENCER, M.D., M.A. Cantab.
F. POOLE LANSDOWN.
L. M. GRIFFITHS.
R. SHINGLETON SMITH, M.D., B.Sc. Lond.
NELSON C. DOBSON.
S. H. SWAYNE.
F. R. CROSS, M.B. Lond.
E. MARKHAM SKERRITT, M.D., B.S., B.A. Lond.
J. GREIG SMITH, M.B., C.M., M.A. Aberd., F.R.S.E.
A. J. HARRISON, M.B. Lond.

J. MICHELL CLARKE, M.D., M.A. Cantab.
JOHN DACRE.
W. H. HARSANT.
B. M. H. ROGERS, M.D., B.Ch., B.A. Oxon.
J. E. SHAW, M.B., C.M. Ed.
G. MUNRO SMITH.

Medical Practitioners wishing to join the Society are requested to communicate with the Honorary Secretary, J. PAUL BUSH, Vyvyan House, Clifton Park, Clifton, Bristol. The Annual Subscription is 21/-.

---

*Extracts from Journal By-laws.*

4.—The *Journal* shall be delivered free to Subscribers and also to Members of the Society whose subscriptions are not in arrear.

6.—The Annual Subscription to the *Journal* for those who pay before the 1st of July shall be 5/-, post free, or 6/- if paid after that date.

---

Communications intended for insertion in the *Journal* should be sent to the Editor, Dr. SHINGLETON SMITH, Deepholm, Clifton Park, Clifton, Bristol.

Books for Review and Exchange Journals should be sent to the Assistant-Editor, L. M. GRIFFITHS, 9 Gordon Road, Clifton, Bristol.

Communications referring to the delivery of the *Journal* should be sent to the Editorial Secretary, Dr. BERTRAM ROGERS, 11 York Place, Clifton, Bristol, to whom Subscriptions are to be paid *in advance.*

Cases for Binding Vols. I.–XIII. are now ready, and may be obtained, price 7*d.* each (postage 2*d.* extra), upon application to J. W. ARROWSMITH, Quay Street, Bristol ; or the *Journal* will be bound, including Case, for 1/3 per volume.

### Bristol Medico-Chirurgical Society.

---

### PAST PRESIDENTS.

1874—75. FREDERICK BRITTAN, M.D., B.A. Dub.

1875—76. ROBERT WILLIAM COE.

1876—77. SAMUEL MARTYN, M.D. Ed.

1877—78. AUGUSTIN PRICHARD, M.D. Berlin.

1878—79. HENRY EDWARD FRIPP, M.D. Würzburg.

1879—80. WILLIAM MICHELL CLARKE.

1880—81. JOSEPH GRIFFITHS SWAYNE, M.D. Lond.

1881—82. EDWARD LONG FOX, M.D. Oxon.

1882—83. JAMES GEORGE DAVEY, M.D. St. And.

1883—84. WILLIAM JOHNSTONE FYFFE, M.D., B.A. Dub.

1884—85. GEORGE FORSTER BURDER, M.D. Aberd.

1885—86. WILLIAM HENRY SPENCER, M.D., M.A. Cantab.

1886—87. FRANCIS POOLE LANSDOWN.

1887—88. LEMUEL MATTHEWS GRIFFITHS.

1888—89. ROBERT SHINGLETON SMITH, M.D., B.Sc. Lond.

1889—90. NELSON CONGREVE DOBSON.

1890—91. SAMUEL HENRY SWAYNE.

1891—92. FRANCIS RICHARDSON CROSS, M.B. Lond.

1892—93. EDWARD MARKHAM SKERRITT, M.D., B.S., B.A. Lond.

1893—94. JAMES GREIG SMITH, M.B., C.M., M.A. Aberd., F.R.S.E.

1894—95. ALFRED JAMES HARRISON, M.B. Lond.

1896.

---

### LIST OF SUBSCRIBERS

TO THE

### Bristol Medico-Chirurgical Journal.

---

*Those marked * are Members of the Society.*

| | |
|---|---|
| Ackland, J. McKno ... ... ... ... ... ... | 24 Southernhay, Exeter. |
| *Ackland, W. R. ... ... ... ... ... ... ... | 5 Rodney Place, Clifton. |
| Adams, George ... ... ... ... ... ... ... | 1 Imperial Road, Redland. |
| Adye, W. J. A. ... ... ... ... ... ... ... ... | Church House, Bradford, Wilts. |
| Aldridge, Charles, M.D., C.M. Aberd.... ... | Plympton. |
| Aldous, George F.... ... ... ... ... ... ... | Charlton House, Mannamead, Plymouth. |
| Alexander, James, M.D., M.Ch., B.A. Dub.... | Bishop's Place, Paignton. |
| Ambrose, J., M.B., C.M. Aberd. ... ... ... ... | 5 Heath Villas, Eastville, Bristol. |
| *Atchley, Cuthbert ... ... ... ... ... ... | 66 Cotham Road, Bristol. |
| Atchley, George F., M.B. Lond. ... ... ... | Tyndall's Park, Bristol. |
| Atkinson, John C., M.B., C.M. Edin. ... ... | 14 Great George Street, Bristol. |
| Avarne, A. B. ... ... ... ... ... ... ... ... | Blaenavon, Monmouthshire. |
| Aveline, H. T. S. ... ... ... ... ... ... ... | Asylum, Stapelton, Gloucestershire. |
| *Ballance, H. Stanley, M.D. Lond. ... ... | 4 Royal Terrace, Weston-super-Mare. |
| Ballantyne, J. W., M.D., C.M. Edin. ... ... | 24 Melville Street, Edinburgh. |
| Bannatyne, Gilbert A., M.D., C.M. Glasg.... | 1 Paragon, Bath. |
| *Barclay, W. M. ... ... ... ... ... ... ... | Queen's Road, Clifton. |
| Baretti, T. G. L'Enardi ... ... ... ... ... | 49 Royal York Crescent, Clifton. |
| *Baron, Barclay J., M.B., C.M. Ed.... ... ... | 16 Whiteladies Road, Clifton. |
| Barr, J. Coleman ... ... ... ... ... ... ... | 2 Cranmore Cottages, Aldershot. |
| *Basset, Walter ... ... ... ... ... ... ... | 24 Stow Hill, Newport, Mon. |
| Batten, Rayner W., M.D. Lond. ... ... ... | 1 Brunswick Square, Gloucester. |
| Batten, W. Smith... ... ... ... ... ... ... | Curzon House, Calne. |
| *Beaver, H. Atwood, M.B., B.Ch.Vict. ... ... | 6 Downleaze Road, Stoke Bishop. |
| *Beddoe, John, M.D.Ed., B.A. Lond., F.R.S. | The Chantry, Bradford-on-Avon. |
| Benham, Harry A., M.D., C.M. Aberd. ... ... | Asylum, Stapleton, Gloucestershire. |
| Bennett, W. S. ... ... ... ... ... ... ... | Escot, Penzance. |
| *Benson, A. H. ... ... ... ... ... ... ... ... | Wrington, Somersetshire. |
| *Berry, F. C., M.D., B.Ch., B.A. Dub. ... ... | Burnham, Somersetshire. |

### LIST OF SUBSCRIBERS.

| | |
|---|---|
| Berry, James, M.B., B.S. Lond. | 60 Welbeck Street, London, W. |
| Bevan, W. L. P., M.D., C.M. Ed. | Alton, Hampshire. |
| Biamonti, Sebastiano | 27 Via Balbi, Genoa, Italy. |
| *Blacker, A. E. | Richmond Hill Avenue, Clifton. |
| *Blagg, Arthur F. | 6 West Mall, Clifton. |
| Blomfield, Arthur G., M.D. Aberd. | 34 West Southernhay, Exeter. |
| *Board, Edmund C. | 11 Caledonia Place, Clifton. |
| Boyce, James G. | The Chipping, Wotton-under-Edge. |
| Boys, Arthur Henry | Chequer Street, St. Alban's. |
| *Brasher, Charles W. J. | 144 Cheltenham Road, Bristol. |
| *Brew, R. H. | Chew Magna. |
| Brimacombe, R. William | Kingswood, Bristol. |
| *Broom, John, M.D. Brux. | St. Paul's Road, Clifton. |
| Brown, G. Arthur | Tredegar. |
| Brown, William, M.D., C.M. Glasg. | Fishponds, Gloucestershire. |
| Browne, J. Walton, M.D., B.A. Royal Univ. I. | 10 College Square North, Belfast. |
| Bullen, F. St. John | 12 Pembroke Road, Clifton. |
| Burroughs, E. F. H. | 3 Elgin Park, Bristol. |
| *Bush, J. Paul | Clifton Park, Clifton. |

| | |
|---|---|
| Callaghan, J. Leslie | Brookfield, Colyton, Devonshire. |
| Campbell, George | Chilton Polden, Somersetshire. |
| *Capron, H. J. | Princes Road, Clevedon. |
| Carolin, G. | Lansdown Road, Dublin. |
| *Carr, Alexander | 150 Wells Road, Bristol. |
| *Carter, T. M. | 3 Clifton Vale, Clifton. |
| *Carter, W. F. | 48 Coronation Road, Bristol. |
| *Carwardine, Thomas, M.B., M.S. Lond. | Royal Infirmary, Bristol. |
| Cave, Edward J., M.D. Lond. | Crewkerne. |
| Cheves, Alexander B., M.D., M.A. Aberd. | Millbrook, Devonshire. |
| Chilton, R. H. | North Point, Durdham Park, Bristol. |
| *Christie, W. L., M.D. Otago, N.Z. | 9 Park Place, Clifton. |
| Church, Basil | Minchinhampton, Gloucestershire. |
| Clapp, George T., M.B. Cantab. | West Southernhay, Exeter. |
| *Clarke, John Michell, M.D., M.A. Cantab. | 28 Pembroke Road, Clifton. |
| Clay, Challoner | Manor House, Fovant. |
| Clay, R. Hogarth, M.D. Ed. | 4 Windsor Villas, Plymouth. |
| Coates, C. J. A. | Chatteris, Cambridgeshire. |
| Cobbold, C. S. W., M.D. Würzburg | Bailbrook House, Bath. |
| *Coe, R. W. | 7 Pembroke Road, Clifton. |
| Coker, T. V. | 2 Chesterfield Place, Clifton. |
| Cole, Thomas, M.D. Lond. | 18 Circus, Bath. |
| *Collins, E. Tenison | 12 Windsor Place, Cardiff. |
| *Collins, H. W. | Wrington, Somersetshire. |
| Colman, Henry | Pewsey, Wiltshire. |
| Colmer, Ptolemy S. H., M.D. Dur. | South Street House, Yeovil. |
| *Combs, Carey, M.D. Lond. | Castle Cary, Somersetshire. |
| *Cook, Henri, M.D., C.M. Aberd. | 45 Stackpool Road, Bristol. |
| Cooke, A. S. | Badbrook House, Stroud. |
| Cory, W. Howard | 1 The Avenue, Redland. |
| Cossham, W. R., M.D., C.M. Aberd. | Cirencester. |
| Cotton, William, M.B., C.M., M.A. Ed. | 227 Gloucester Road, Bristol. |
| Cousins, J. Ward, M.D. Lond. | Kent Road, Southsea. |
| Cowan, F. S. | 27 Queen Square, Bath. |
| Craddock, Samuel | South Lyncombe, Bath. |
| Crallan, G. E., M.A., M.B. Cantab. | Brislington House, Brislington. |
| Crespi, Alfred J. H. | Poole Road, Wimborne. |
| *Cross, F. Richardson, M.B. Lond. | Worcester House, Clifton. |

| | |
|---|---|
| *Crossman, Edward, M.D. Dur. | Hambrook, Gloucestershire. |
| Crouch, C. Percival, M.B. Lond. | 3 Princes Buildings, Weston-super-Mare. |
| Culross, James, M.B., C.M., M.A. Glasg. | 66 Queen Street, Newton Abbot. |
| Cunningham, C. L. | Chudleigh. |
| | |
| *Dacre, John | 14 Eaton Crescent, Clifton. |
| Dalby, Augustus W. | Redland House, Frome. |
| Daniel, T. P. | Beaminster, Dorsetshire. |
| *Davies, D. S., M.D. Lond., D.P.H. Cantab. | 60 Oakfield Road, Clifton. |
| Davies, Ebenezer | Brunswick House, Swansea. |
| Davies, H. N. | Porth, Glamorganshire. |
| Davis, Theodore, M.D. Lond. | Beachcroft, Clevedon. |
| Davy, Henry, M.D. Lond. | Southernhay House, Exeter. |
| Day, Percy Howard | Stalmine, Poulton-le-Fylde. |
| Dening, Edwin | Manor House, Stow-on-the-Wold. |
| Devis, Harry F. | Wells Road, Bristol. |
| Dickie, James Steel, M.D., C.M. Aberd. | Boscombe Park, Bournemouth. |
| *Dobson, Nelson C. | 27 Victoria Square, Clifton. |
| Domville, Edward J. | 44 Southernhay, Exeter. |
| *Dowson, W., M.B., B.C., M.A. Cantab. | 17 Clyde Park, Bristol. |
| Doyne, Robert W. | St. Giles', Oxford. |
| Dunbar, Eliza L. Walker, M.D. Zürich | 9 Oakfield Road, Clifton. |
| | |
| Eadon, W. F. Bailey | Hambrook, Gloucestershire. |
| *Eager, Reginald, M.D. Lond. | Northwoods, Winterbourne. |
| Eddowes, Charles | Maddington, Devizes. |
| *Edgeworth, F. H., M.B., B.C., B.A. Cantab., B.Sc. Lond. | 1 Richmond Hill, Clifton. |
| Edwards, W. T., M.D. Lond. | 129 Queen Street, Cardiff. |
| Elder, William | 7 Trelawney Road, Bristol. |
| *Elliott, Christopher, M.D., B.A. Dub. | 88 Pembroke Road, Clifton. |
| Ellis, W. Mc D., M.D. Brussels | 7 Alexander Buildings, Bath. |
| Embleton, Dennis C. | St. Michael's Road, Bournemouth. |
| Etches, W. R. | Macclesfield. |
| Evans, J. Fenton, M.B., C.M. Ed. | 6 Whitehall Yard, London, S.W. |
| Evans, T. G. C. | Budleigh Salterton, Devon. |
| *Ewens, John | The Elms, Cotham Hill, Bristol. |
| | |
| Fairbanks, W., M.D., C.M. Ed. | Wells, Somersetshire. |
| *Fawcett, E., M.B., C.M. Ed. | 141 Redland Road, Bristol. |
| *Fendick, R. George | St. Paul's Road, Clifton. |
| Fenwick, Charles | Dunsford, Exeter. |
| Fergusson, W. Balfour, M.D., C.M. Ab. | Hazelbury House, Painswick. |
| Finch, Thomas, M.B., B.A. Cantab. | Watcombe View, Babbacombe. |
| *Firth, J. L., M.D., B.S. Lond. | General Hospital, Bristol. |
| *Fisher, Theodore, M.D. Lond. | 25 Pembroke Road, Clifton. |
| *Flemming, C. E. S. | Freshford. |
| Ford, Joseph | Wedmore. |
| Forty, D. H. | Wotton-under-Edge. |
| Fowler, O. H. | Cirencester. |
| Fox, Arthur E. W., M.B., C.M. Ed. | 16 Gay Street, Bath. |
| *Fox, Bonville B., M.D., M.A. Oxon. | Brislington. |
| *Fox, E. Long, M.D. Oxon. | Church House, Clifton. |
| Fox, John Alfred | Tregea House, Penzance. |
| Fox, T. Colcott, M.B. Lond., B.A. Cantab. | 14 Harley Street, London, W. |
| Fraser, Græme B. | Beach Road, Weston-super-Mare. |
| Freeman, Henry W. | 24 Circus, Bath. |

| | |
|---|---|
| *Freeman, J. | Cheltenham Road, Bristol. |
| Frood, James N. | Topsham, Devon. |
| *Fuller, Joseph | Long Ashton. |
| *Fyffe, W. Johnstone, M.D., B.A. Dub. | 2 Rodney Place, Clifton. |
| Gamble, C. H. | Barnstaple. |
| *Garland, E. B., M.B., C.M. Edin. | 92 Hampton Road, Redland. |
| Garner, John E., M.D., C.M. Aberd. | 6 Wenckley Square, Preston, Lancashire. |
| Genge, T. T. | 21 Whiteladies Road, Bristol. |
| *Gerrish, D. G. | Royal Infirmary, Bristol. |
| *Gibbs, Alfred N. Godby | 52 Whiteladies Road, Clifton. |
| Gidley, G. G. | Cullompton, Devon. |
| Gloag, George A. | 7 College Green, Bristol. |
| Goodfellow, W. R. | Roche, Cornwall. |
| Goodridge, Henry F. A., M.D. Lond. | 10 Brock Street, Bath. |
| Goodwyn, A. H. | Droxford, Hampshire. |
| Goss, T. Biddulph | 1 Circus, Bath. |
| Grace, Alfred | Chipping Sodbury. |
| Grace, Edward Mills | Thornbury, Gloucestershire. |
| Grace, W. G. | 15 Victoria Square, Clifton. |
| Gratte, C. Brooke | 3 Lansdown Place, Newport, Mon. |
| *Gray, A. Murray | 17 Market Place, Devizes. |
| Greenway, A. G., M.D. R.U.I. | Llandrindod Wells, Radnorshire. |
| Grey, T. C. | Church House, St. Neot's. |
| *Griffiths, J. S. | 25 Redland Park, Bristol. |
| *Griffiths, L. M. | 9 Gordon Road, Clifton. |
| Griffiths, P. Rhys, M.B., B.S. Lond. | 71 Newport Road, Cardiff. |
| Groves, J., M.B., B.A. Lond. | Carisbrooke. |
| Hadwen, W. R. | Highbridge, Somersetshire. |
| *Hailes, Clements D. G., M.D., C.M. Ed. | 27 Alma Road, Clifton. |
| *Haines, A. H. | 8 Cotham Road, Bristol. |
| Hale, Edmund T. | The Grange, Chew Magna. |
| Halliwell, T. O. | Blagdon, Somerset. |
| Halpin, W. C. | Parkend, Gloucestershire. |
| Hamlen-Williams, T. R. | Verlands, Cowbridge, Glamorganshire. |
| Handfield-Jones, Montagu, M.D. Lond. | 35 Cavendish Square, London, W. |
| Hardwick, A., M.B. Dur. | Newquay, Cornwall. |
| Harper, Joseph | Bear Street, Barnstaple. |
| *Harrison, A. J., M.B. Lond. | Guthrie Road, Clifton. |
| Harrison, Charles | Keynsham, Somersetshire. |
| Harsant, J. G., M.D., B.S. Lond. | Exeter Road, Bournemouth. |
| *Harsant, W. H. | Tower House, Clifton. |
| *Harvey, Alfred, M.B. Lond. | Westbury-on-Trym. |
| Hawkins-Ambler, G. A. | 162 Upper Parliament Street, Liverpool. |
| Hawkins, Cæsar F. | North Petherton. |
| Hayman, Alfred G. | 1 Pembroke Road, Clifton. |
| *Hayman, C. A. | 19 Hanbury Road, Clifton. |
| *Heaven, John C., D.Ph. Lond. | 17 Whiteladies Road, Bristol. |
| Hemming, J. Hughes | Kimbolton, St. Neot's. |
| *Herapath, C. K. C. | Cotham Park, Bristol. |
| *Hetling, H. E. | 30 Elliston Road, Bristol. |
| Hill, Hedley | 1 Redcliff Hill, Bristol. |
| *Hill, Thomas, M.D. St. And. | 138 Cromwell Road, Bristol. |
| *Hill, Walter J. | Edgcumbe Villa, Clevedon. |
| Hoblyn, Francis Parker | 10 Bladud Buildings, Bath. |
| Hoffman, A. W. W. | 30 The Parade, Leamington. |

| | |
|---|---|
| HOLLINGS, EDWIN T. ... ... ... ... ... ... | Woolacombe, Morthoe. |
| HORSFALL, JOHN, M.A. Oxon. ... ... ... ... | Richmond Hill, Bournemouth. |
| HOSPITAL, BRISTOL GENERAL (*Sec.*, W. THWAITES). | |
| HOSPITAL, TAUNTON AND SOMERSET (*House-Surgeon*, J. G. BAIN). | |
| HOUGHTON, LEONARD F. ... ... ... ... ... | East Looe, Cornwall. |
| HOWELLS, WILLIAM, M.B., C.M. Glasg. ... ... | Church House, Talgarth, Breconshire. |
| HOWSE, WILLIAM ... ... ... ... ... ... ... ... | 8 London Street, Swindon, Wiltshire. |
| HOWSIN, E. ARTHUR, M.D. St. And. ... ... ... | Stroud. |
| HUDSON, F. HORACE ... ... ... ... ... ... | Sturminster Newton. |
| HUDSON, H. R. ... ... ... ... ... ... ... ... | 182 Commercial Road, Newport, Mon. |
| HUGGARD, W. R., M.D., M.Ch., M.A. Roy. Univ. I. | Davos-Platz, Switzerland. |
| HUMPHREYS, HENRY, M.D., M.A. Cantab. ... | St. Mary Church Road, Torquay. |
| HUNT, A. D. ... ... ... ... ... ... ... ... | Millbrook House, Chagford. |
| HYATT, J. T. ... ... ... ... ... ... ... ... | Shepton Mallet. |
| IMLAY, G. A., M.B., C.M. Aberd. ... ... ... | 1 Cotham Park, Bristol. |
| INSTITUTION, LIVERPOOL MEDICAL (*Resident Librarian*, WILLIAM JONES). | |
| IVES, WILLIAM R. Y. ... ... ... ... ... ... | Portswood Road, Southampton. |
| JACKSON, MARK, M.D. Roy. Univ. I. ... ... ... | Bear Street, Barnstaple. |
| JACOBSON, W. H. A., M.B., M.C., M.A. Oxon. | 66 Great Cumberland Place, London, W. |
| JAMES, J. BOWEN, ... ... ... ... ... ... ... | Woodlands, Aberaman, Aberdare. |
| JAMISON, A., M.B., B.Ch., M.A. Roy. Univ. I. ... | Belfast. |
| JESSOP, CHARLES MOORE ... ... ... ... ... | Clare Lodge, Redhill. |
| JOHNSON, G. H.... ... ... ... ... ... ... ... | Teignmouth. |
| JONES, A. ORLANDO, M.D., C.M. Aberd. ... ... | Harrogate. |
| JONES, EVAN ... ... ... ... ... ... ... ... | Ty-mawr, Aberdare. |
| JONES, H. MACNAUGHTON, M.D., M.Ch. Roy. U.I. | 141 Harley Street, London, W. |
| JONES, W. W., M.B., C.M. Ed. ... ... ... ... | 47 Wellington Street, Merthyr Tydvil. |
| *KELLY, T. B. ... ... ... ... ... ... ... ... | Eye Hospital, Bristol. |
| *KEMM, F. ST. J.... ... ... ... ... ... ... ... | Worle. |
| KENDALL, BERNARD C. ... ... ... ... ... ... | Elm Grove, Penally, Pembrokeshire. |
| *KENDALL, H. W. ... ... ... ... ... ... ... | 9 King's Parade, Clifton. |
| KENNEDY, W. P., M.D., B.Ch., B.A. Dub. ... | High Street, Lydney. |
| *KING, PRESTON, M.D. Cantab. ... ... ... ... | 29 Gay Street, Bath. |
| KINNEIR, R. ... ... ... ... ... ... ... ... | The Tower House, Malmesbury. |
| *LACE, F. ... ... ... ... ... ... ... ... ... | 5 Paragon, Bath. |
| LANCASTER, E. LE CRONIER, B.A., M.B., B.Ch. Oxon. ... ... ... ... ... ... ... ... | Winchester House, Swansea. |
| LANGRIDGE, F. W. ... ... ... ... ... ... ... | Croftside, Ilfracombe. |
| *LANSDOWN, F. POOLE ... ... ... ... ... ... | 19 Whiteladies Road, Clifton. |
| *LANSDOWN, R. G. P., M.B., B.S. Dur. ... ... | 39 Oakfield Road, Clifton. |
| LAUWERS, DR. ... ... ... ... ... ... ... ... | Courtrai, Belgium. |
| *LAWRENCE, A. E. AUST, M.D., C.M. Aberd. ... | 19 Richmond Hill, Clifton. |
| LAWRIE, J. MACPHERSON, M.D., C.M. Glasg.... | Greenhill, Weymouth. |
| LEACH, J. COMYNS, M.D. Dur., B.Sc. Lond. ... | Sturminster Newton. |
| *LEES, E. LEONARD, M.D., C.M. Ed. ... ... ... | Redland Road, Bristol. |
| LEIGH, W. W. ... ... ... ... ... ... ... ... | Treharris, Glamorganshire. |
| LEWELLYN, J. ... ... ... ... ... ... ... ... | Caerphilly. |
| LIDIARD, S. A. ... ... ... ... ... ... ... ... | Kimberley Road, Falmouth. |
| LIDDERDALE, JAMES ... ... ... ... ... ... | Henley-on-Thames. |
| *LIGERTWOOD, WALTER ... ... ... ... ... ... | Altona, Elmdale Road, Clifton. |

| | |
|---|---|
| LINDSAY, JAMES A., M.D., M.Ch., M.A.Roy.U.I. | 37 Victoria Place, Belfast. |
| LISTER, SIR JOSEPH, Bart., F.R.S. ... ... ... | 12 Park Crescent, London, N.W. |
| *LOWE, T. PAGAN ... ... ... ... ... ... ... | 16 Circus, Bath. |
| LUCY, REGINALD H., M.B., C.M. Ed. ... ... | 9 Princes Square, Plymouth. |
| | |
| *MCCREA, P. W., M.D., B.Ch., B.A., B.A.O., B.A. Dub. ... ... ... ... ... ... ... ... | 19 Portland Square, Bristol. |
| MACDONALD, J. A., M.D., M.Ch. Roy. Univ. I. | 19 East Street, Taunton. |
| MAC DONALD, P. W., M.D., C.M. Aberd. ... ... | County Asylum, Dorchester. |
| MAC DOUGALL, HENRY R. ... ... ... ... ... | Shepscombe, Stroud. |
| MCELFATRICK, JOHN ... ... ... ... ... ... | The Chantry, Mere, Wiltshire. |
| MAC ILWAINE, S. W. ... ... ... ... ... ... | South Petherton, Somersetshire. |
| *MACKAY, H. J., M.B., C.M. Ed. ... ... ... ... | 10 Long Street, Devizes. |
| MAC KEITH, JOHN, M.B., C.M. Glasg. ... ... | St. Andrew, The Barnfield, Exeter. |
| MACKIE, J., M.B., LL.D. Aberd., C.M.G. ... | Alexandria, Egypt. |
| MACKINNON, CHARLES, M.B., C.M., M.A. Glasg. | Dyer Street, Cirencester. |
| MACLEAN, EWEN J., M.D., C.M. Ed.... ... ... | 51 Linden Gardens, London, W. |
| MACLEAN, J. CAMPBELL, M.B., C.M. Ed.... ... | Swindon House, Swindon, Wiltshire. |
| MACREADY, J. ... ... ... ... ... ... ... ... | 51 Queen Ann Street, London, W. |
| MARSH, O. E. BULWER ... ... ... ... ... ... | Parkdale, Newport, Mon. |
| MARSHALL, ARTHUR L., M.B., B.A. Cantab. ... | 2 Clapton Common, London. |
| *MARSHALL, HENRY, M.D. Ed., F.R.S.E. ... ... | 28 Caledonia Place, Clifton. |
| *MARTIN, E. F., M.B. Ed. ... ... ... ... ... | Victoria House, Weston-super-Mare. |
| MASON, S. B. ... ... ... ... ... ... ... ... | 12 Park Terrace, Pontypool. |
| MASON, WILLIAM ... ... ... ... ... ... ... | Sedgemoor Cottage, St. Austell. |
| *MATTHEWS, CHARLES E. ... ... ... ... ... | 51 Pembroke Road, Clifton. |
| MAYOR, T. O. ... ... ... ... ... ... ... ... | 40 Apsley Road, Clifton. |
| MEREDITH, JOHN, M.D. Ed. ... ... ... ... ... | Wellington, Somersetshire. |
| MILLARD, W. J. KELSON, M.D. St. And. ... ... | Arima, Torquay. |
| *MOLE, HAROLD F. ... ... ... ... ... ... ... | Royal Infirmary, Bristol. |
| MONCKTON, WILLIAM ... ... ... ... ... ... | Portishead. |
| MORGAN, JOHN ... ... ... ... ... ... ... ... | Langport, Somersetshire. |
| *MORGAN, T. WHITWORTH S. ... ... ... ... | Pill. |
| *MORTON, C. A. ... ... ... ... ... ... ... ... | 14 Vyvyan Terrace, Clifton. |
| MOUAT-BIGGS, C. E. F.... ... ... ... ... ... | 2 Fauconberg Villas, Cheltenham. |
| MUNDEN, CHARLES ... ... ... ... ... ... ... | Ilminster. |
| MURRAY, WILLIAM, M.B., C.M. Ed. ... ... ... | Staple Hill, Gloucestershire. |
| *MYLES, G. T. ... ... ... ... ... ... ... ... | 3 Portland Square, Bristol. |
| | |
| NEALE, B. G. ... ... ... ... ... ... ... ... | 51 Upper Belgrave Road, Clifton. |
| NELL, RICHARD F. ... ... ... ... ... ... ... | 33 Windsor Terrace, Penarth. |
| *NEVILL, W. N., M.D., B.Ch., B.A. Dub. ... ... | Southville, Bristol. |
| *NEWMAN, CHARLES ... ... ... ... ... ... ... | 156 Cheltenham Road, Bristol. |
| *NEWNHAM, W. H. C., M.B., M.A. Cantab. ... | 1 Leicester Place, Clifton. |
| NICHOLSON, T. D., C.M. Ed. ... ... ... ... | 2 Whiteladies Road, Clifton. |
| NORMAN, J. H. ... ... ... ... ... ... ... ... | Goodleigh, Winkleigh, Devonshire. |
| *NORTON, J. A., M.D., C.M. Aberd. ... ... ... | 65 Redcliff Hill, Bristol. |
| | |
| O'BRIEN, CORNELIUS ... ... ... ... ... ... | Down House, Whitehall, Bristol. |
| ODELL, WILLIAM ... ... ... ... ... ... ... | Ferndale, Torquay. |
| *OGILVY, ALEXANDER, M.D., B.Ch., B.A. Dub. | 20 Pembroke Road, Clifton. |
| OPENSHAW, E. HYDE ... ... ... ... ... ... | Barrules, Cheddar. |
| ORMEROD, HENRY ... ... ... ... ... ... ... | Westbury-on-Trym. |
| *ORMEROD, H. LAWRENCE, M.B., B.Ch., B.A.O., R.U.I. ... ... ... ... ... ... ... ... | Foxstones, Westbury-on-Trym. |

| | |
|---|---|
| PAGE, G. SHEPLEY | 78 Old Market Street, Bristol. |
| *PARKER, GEORGE, M.D., M.A. Cantab. | 14 Pembroke Road, Clifton. |
| PARRY, J. H. | 199 Cheltenham Road, Bristol. |
| PARSONS, FRANCIS J. C. | King Square, Bridgwater. |
| PATERSON, DONALD ROSE, M.D., C.M. Ed. | 18 Windsor Place, Cardiff. |
| PEAKE, ARTHUR W. | 1 Bridewell Street, Bristol. |
| PEAKE, G. ARTHUR | Rodney Place, Cheltenham. |
| *PENNY, W. J. | Coombe, West Crewkerne, Somerset. |
| PHILLIPS, EDWARD PICTON | High Street, Haverfordwest. |
| PHILLIPS, J. A. | 72 Carlisle Street, Cardiff. |
| PHILLIPS, SUTHERLAND REES, M.D., M.Ch. Roy. Univ. I. | St. Anne's Heath, Virginia Water, Surrey. |
| *PICKERING, C. F. | 12 Berkeley Square, Bristol. |
| *PINCH, A. E. H. | 53 Oakfield Road, Clifton. |
| PLAISTER, W. H. | High Road, Tottenham. |
| POLLARD, REGINALD, M.B. Dur. | Southlands, Torquay. |
| *POUNTNEY, W. E., M.D., C.M. Ed. | 33a Whiteladies Road, Bristol. |
| POWELL, J. J., M.D. Lond. | 2 Portland Square, Bristol. |
| POWELL, WILLIAM, M.B. Lond. | Hill Garden, Torquay. |
| POWER, CHARLES J. | Hazelwood, Nailsworth. |
| PRICE, WILLIAM, M.B. Lond. | 40 Park Place, Cardiff. |
| *PRICHARD, ARTHUR W. | 6 Rodney Place, Clifton. |
| *PRICHARD, AUGUSTIN, M.D. Berlin | 4 Chesterfield Place, Clifton. |
| *PRICHARD, J. E., M.B., B.A. Oxon. | 243 Cheltenham Road, Bristol. |
| *PROWSE, ARTHUR B., M.D. Lond. | 5 Lansdown Place, Clifton. |
| PULLIN, THOS. H. S., M.D. | Sidmouth. |
| PURDY, J. R., M.D. Aber. | Lower Hutt, New Zealand. |

| | |
|---|---|
| RANDOLPH, CHARLES | St. Michael's, Milverton, Somersetshire. |
| *RATTRAY, J. M., M.D., C.M., M.A. Aberd. | Frome. |
| REDWOOD, T. HALL, M.D., C.M., M.A. Dur. | The Lawn, Rhymney, Monmouthshire. |
| REES, ALFRED | 46 London Square, Cardiff. |
| RENDALL, JOHN | Forestside, Lymington. |
| *RICHARDSON, W., M.D. St. And. | 1 Belgrave Villas, Cotham Grove, Bristol. |
| RIDDELL, ANDREW W. | Hurstbourne Tennant, Andover. |
| ROBATHAN, G. B. | The Grove, Risca. |
| ROBERTS, FREDERICK T., M.D., B.Sc. Lond. | 102 Hartley Street, London, W. |
| RODERICK, SYDNEY J., M.B., C.M. Ed. | Llanelly. |
| RODGERS, JOHN W., M.B., C.M. Ed. | Redfield, Gloucestershire. |
| *ROGERS, B. M. H., M.D., B.Ch., B.A. Oxon. | 11 York Place, Clifton. |
| *ROSSITER, GEORGE F., M.B. Lond. | Cairo Lodge, Weston-super-Mare. |
| *ROUÉ, W. BARRETT, M.D., M.S. Dur. | 2 Buckingham Place, Clifton. |
| ROUTH, R. HENRY F. | Bridgewater. |
| ROW, F. EVERARD | 9 Tamar Terrace, Devonport. |
| *ROXBURGH, ROBERT, M.B. Ed. | 1 Victoria Buildings, Weston-super-Mare. |
| *RUDGE, C. KING | 145 Whiteladies Road, Bristol. |
| *RUDGE, H. T. | 5 Colston Parade, Bristol. |
| RUFFER, M. ARMAND, M.D., B.S., M.B. Oxon. | Cairo, Egypt. |
| RUTHERFORD, H. T. | Park Street, Taunton. |

| | |
|---|---|
| SCOTT, JAMES, M.B., C.M. Ed. | 24 Gaolgate Street, Stafford. |
| SCOTT, RICHARD, J. H. | 28 Circus, Bath. |
| SEARLE, GEORGE C. | Brixham, Devonshire. |
| SEARLE, RICHARD BURFORD | Bexley, Dartmouth. |
| SEMPLE, H. F. | Budleigh Salterton, Devonshire. |
| SEMPLE, W. R. M., M.B., C.M. Glasg. | Henford Park, Yeovil, Somersetshire. |

### LIST OF SUBSCRIBERS.

| | |
|---|---|
| SEWARD, W. J., M.B. Lond. ... ... ... ... | Asylum, Colney Hatch, London, N. |
| *SHAW, J. E., M.B., C.M. Ed. ... ... ... ... | 23 Caledonia Place, Clifton. |
| SHAW, JESSE, M.D.... ... ... ... ... ... ... | Fort Beaufort, Cape Colony. |
| SHEEN, A., M.D. St. And. ... ... ... ... ... | 23 Newport Road, Cardiff. |
| *SHEPPARD, E. J. ... ... ... ... ... ... ... | 3 Buckingham Place, Clifton. |
| SHORTRIDGE, THOMAS WOOD, M.D. Brussels | Honiton. |
| SIMONS, C. E. G., M.B., C.M. Aberd. ... ... | The Hollies, Merthyr Tydvil. |
| SKELTON, HENRY, M.D. St. And. ... ... ... | Downend, Gloucestershire. |
| *SKERRITT, E. MARKHAM, M.B., B.S., B.A. Lond. | Richmond Hill, Clifton. |
| *SMITH, G. MUNRO ... ... ... ... ... ... ... | 28 Alma Road, Clifton. |
| *SMITH, J. GREIG, M.B., C.M., M.A. Ab., F.R.S.E. | 16 Victoria Square, Clifton. |
| *SMITH, J. WESLEY, M.B., C.M. Aberd. ... ... | Boulevard, Weston-super-Mare. |
| SMITH, REV. REGINALD, M.A. Cantab. ... ... | Walpole St. Andrew's, Wisbeach. |
| *SMITH, R. SHINGLETON, M.D., B.Sc. Lond. ... | Clifton Park, Clifton. |
| SMITH, W. ALEXANDER, M.B., M.A. Oxon. ... | Newport, Essex. |
| SOCIETY, CARDIFF MEDICAL (*Hon. Sec.*, DR. D. R. PATERSON). | |
| SOCIETY, COLUMBIAN MEDICAL ... ... ... ... | 130 State Street, Chicago, U.S.A. |
| SOCIETY, PLYMOUTH MEDICAL (*Hon. Lib.*, C. E. R. RENDLE). | |
| SOCIETY, TORQUAY MEDICAL (*Hon. Sec.*, G. YOUNG EALES). | |
| SOLLY, REGINALD V., M.B., B.S. Lond. ... ... | 40 Southernhay, Exeter. |
| SOLLY, S. E. ... ... ... ... ... ... ... ... | Colorado Springs, Colorado, U. S. A. |
| SOMER, JAMES ... ... ... ... ... ... ... ... | Broad Clyst. |
| SOUTAR, JAMES GREIG, M.B., C.M. Ed. ... ... | Barnwood House, Gloucester. |
| SPENDER, J. KENT, M.D. Lond. ... ... ... ... | 9 Circus, Bath. |
| STAMP, W. D. ... ... ... ... ... ... ... ... | Ridgeway House, Plympton. |
| STATHAM, R. WHITESIDE ... ... ... ... ... | Cheddar, Somersetshire. |
| *STEELE, CHARLES, M.D. Dur. ... ... ... ... | 17 Richmond Hill, Clifton. |
| STEELE-PERKINS, E. B. ... ... ... ... ... ... | 1 Hill's Buildings, Exeter. |
| STEVENS, W. H. ... ... ... ... ... ... ... ... | Zetland Road, Bristol. |
| STODDART, F. WALLIS ... ... ... ... ... ... | 1 Park Street, Bristol. |
| *SWAIN, JAMES, M.D., M.S. Lond. ... ... ... | 7 Buckingham Place, Clifton. |
| *SWAYNE, J. G., M.D. Lond. ... ... ... ... ... | 74 Pembroke Road, Clifton. |
| *SWAYNE, S. H. ... ... ... ... ... ... ... ... | 129 Pembroke Road, Clifton. |
| *SWAYNE, W. CARLESS, M.D. Lond. ... ... ... | 8 Leicester Place, Clifton. |
| SWETE, H. LAWTON ... ... ... ... ... ... | Fishguard, Pembrokeshire. |
| SYMONDS, H. P. ... ... ... ... ... ... ... ... | 35 Beaumont Street, Oxford. |
| SYMONS, JOHN ... ... ... ... ... ... ... ... | Buriton House, Penzance. |
| | |
| TAYLOR, C. BELL, M.D. Edin. ... ... ... ... | 9 Park Row, Nottingham. |
| *TAYLOR, JAMES ... ... ... ... ... ... ... ... | 36 Alma Road, Clifton. |
| TAYLOR, THOMAS HENRY ... ... ... ... ... | Thornbury, Gloucestershire. |
| THOMAS, A. GARROD, M.D., C.M. Ed. ... ... | Clytha Park, Newport, Mon. |
| THOMAS, J. LYNN ... ... ... ... ... ... ... | 28 Charles Street, Cardiff. |
| THOMAS, JOHN T. ... ... ... ... ... ... ... | Brynhyfryd, Chelston, Torquay. |
| THOMAS, ROWLAND ... ... ... ... ... ... ... | The Cwfr, St. Clears, Carmarthenshire. |
| THOMAS, W. H.... ... ... ... ... ... ... ... | Maesteg, Glamorganshire. |
| THOMSON, EUSTACE B., M.D., C.M. Glasg. ... | Albany Place, Plymouth. |
| THOMPSON, W. C. ... ... ... ... ... ... ... | 189 Cheltenham Road, Bristol. |
| *TIVY, W. J.... ... ... ... ... ... ... ... | 8 Lansdown Place, Clifton. |
| TOLPUTT, PERCY T... ... ... ... ... ... ... | Weaver Lane, Northwich. |
| TOSSWILL, LOUIS H., M.B., B.A. Cantab.... ... | 28 West Southernhay, Exeter. |
| TRACY, H. E., M.B. Lond. ... ... ... ... ... | Willand. |
| TREVITHICK, EDGAR, M.A., M.D. Cantab. ... | 24 Promenade, Cheltenham. |
| TROTTER, LESLIE B., M.D., C.M. Ed. ... ... | Coleford, Gloucestershire. |
| TYLEY, R. P., M.D. St. And.... ... ... ... ... | Wedmore. |
| | |
| VACHELL, HERBERT R., M.D., C.M. Aberd. ... | 18 Newport Road, Cardiff. |
| VEREKER, R. H. ... ... ... ... ... ... ... | Eastleigh, Curry Rivel. |

### LIST OF SUBSCRIBERS.

| | |
|---|---|
| Wade, A. Law, M.D., B.A. Dub. | County Asylum, Wells, Somersetshire. |
| Wade, Charles H., M.A. Oxon. | Stanfield, Torquay. |
| *Waldo, Henry, M.D., C.M. Aberd. | 19 Pembroke Road, Clifton. |
| *Walker, Cyril H., M.B., B.A. Cantab. | St. Paul's Road Clifton. |
| Wallace, Rev. Canon, M.A. Oxon. | 3 Harley Place, Clifton. |
| *Wallace, John | 1 Oriel Terrace, Weston-super-Mare. |
| Walsh, Leslie H. | Royal United Hospital, Bath. |
| Ward, J. L. W. | Victoria Street, Merthyr Tydvil. |
| *Wathen, J. Hancocke | 16 York Place, Clifton. |
| Watters, George T. B., M.D., C.M. Ed. | Stonehouse, Gloucestershire. |
| Waugh, Alexander | Midsomer Norton. |
| Weatherly, Lionel A., M.D., C.M. Aberd. | Bailbrook House, Bath. |
| Webber, William W. | Crewkerne. |
| Webster, Thomas | Redland Hill, Bristol. |
| Wedmore, C. Ernest, M.B., M.A. Cantab. | Stoke Bishop. |
| Wethered, Frank J., M.D. Lond. | 83 Harley Street, London, W. |
| *Whicher, A. H. | Midsomer Norton. |
| Wickham, W. | Hill House, Tetbury. |
| Wigan, C. A., M.D. Dur. | Portishead. |
| Wigmore, J., M.D. Roy. Univ. I. | 20 Green Park, Bath. |
| Wilcox, Henry, M.B. Aberd. | Fleet, Hampshire. |
| Wilde, Rowland, M.B. Edin. | Park House, Weston-super-Mare. |
| *Wilding, James, M.D. Dur. | Sydenham Road, Bristol. |
| Willcox, R. Lewis | Warminster. |
| Willett, George G. D. | Keynsham, Somersetshire. |
| Williams, Charles | 118 Derby Road, Bootle, Liverpool. |
| Williams, E. C., M.B. Cantab. | Children's Hospital, Bristol. |
| Williams, H. Egerton | The Mount, Newport, Mon. |
| Williams, John, M.D. | 20 Windsor Place, Cardiff. |
| *Williams, Lionel H. | Thornbury, Gloucestershire. |
| *Williams, P. Watson, M.D. Lond. | 2 Lansdown Place, Clifton. |
| Williams, Peter | Ferryside, Carmarthenshire. |
| *Willis, W. M. | Children's Hospital, Bristol. |
| *Windsor-Aubrey, H. W. | Coronation Road, Bristol. |
| Winterbotham, L. | Bays Hill, Cheltenham. |
| *Wintle, Colston | 24 Gloucester Road, Bristol. |
| Woodford, E. Russell, M.D., C.M. Ed. | 4 Prospect Terrace, Ventnor. |
| Woodhead, G. Sims, M.D., C.M. Ed. | 1 Nightingale Lane, London, S.W. |
| Woods, Frank | Timsbury. |
| Woollcombe, Walter L. | 16 Lockyer Street, Plymouth. |

### Exchange-List.

### AMERICA.

### ARGENTINE REPUBLIC.

*Weekly.*

*La Semana Médica.* Buenos Ayres: J. A. Manzano.

### CANADA.

*Monthly.*

*The Canadian Practitioner.* Toronto: The Bryant Press.

*The Montreal Medical Journal.* Montreal: Gazette Printing Company.

### MEXICO.

*Twice a month.*

*Gaceta Médica.* Mexico: Avenida 2 Oriente, 726.

### PERU.

*Twice a month.*

*La Crónica Médica.* Lima: 24 Calle de Bodegones.

### UNITED STATES.

*Weekly.*

*Boston Medical and Surgical Journal.* Boston: Damrell & Upham.

*Medical Record.* New York: William Wood & Co.

*The Cincinnati Lancet-Clinic.* Cincinnati: 317 West 7th Street.

*The Journal of the American Medical Association.* Chicago: 86 Fifth Avenue.

*The Medical and Surgical Reporter.* Philadelphia: The Butler Publishing Company.

*The Medical News.* New York: Lea Brothers & Co.

*The New York Medical Journal.* New York: D. Appleton & Co.

*Fortnightly.*

*The American Practitioner and News.* Louisville: John P. Morton and Company.

*Twice a month.*

*Pediatrics.* New York: Van Publishing Co.

*The Medical Age.* Detroit: George S. Davis.

*Monthly.*

*Archives of Pediatrics.* New York: E. B. Treat.

*Buffalo Medical Journal.* Buffalo: A. T. Brown Printing House.

*Bulletin of the Johns Hopkins Hospital.* Baltimore: The Johns Hopkins Press.

*International Medical Magazine.* Philadelphia: International Medical Magazine Company.

*Journal of Cutaneous and Genito-Urinary Diseases.* New York: D. Appleton & Co.

*Occidental Medical Times.* Sacramento: D. Johnston & Co.

*The American Gynæcological and Obstetrical Journal.* New York: D. Appleton and Company.

*The American Journal of Obstetrics and Diseases of Women and Children.* New York: William Wood & Co.

*The American Journal of Ophthalmology.* St. Louis: 3036 Locust Street.

*The American Journal of the Medical Sciences.* Philadelphia: Lea Brothers & Co.

*The Journal of Nervous and Mental Disease.* New York: 25 West 45th Street.

*The Post-Graduate.* New York: 2nd Avenue and 20th Street.

*The Saint Louis Medical and Surgical Journal.* Saint Louis: St. Louis Medical and Surgical Journal Publishing Company.

*The Therapeutic Gazette.* Detroit: George S. Davis.

*The Universal Medical Journal.* Philadelphia: The F. A. Davis Co.

*University Medical Magazine.* Philadelphia: University of Pennsylvania Press.

*Quarterly.*

*Mathews' Medical Quarterly.* Louisville: John P. Morton & Company.

*The Alienist and Neurologist.* St. Louis: Ev. E. Carreras.

*Yearly.*

*Annual of the Universal Medical Sciences.* Philadelphia: The F. A. Davis Company.

*The American Year-Book of Medicine and Surgery.* Philadelphia: W. B. Saunders.

*Transactions of the American Dermatological Association.* New York: Geo. L. Goodman & Co.

*Transactions of the American Laryngological Association.* New York: D. Appleton and Company.

*Transactions of the American Surgical Association.* Philadelphia: William J. Dornan.

*Transactions of the Association of American Physicians.* Philadelphia: William J. Dornan.

*Transactions of the College of Physicians of Philadelphia.* Philadelphia: William J. Dornan.

*Transactions of the Colorado State Medical Society.* Denver: A. J. Ludditt.

*Transactions of the Iowa State Medical Society.* Des Moines: The Kenyon Printing and Mfg. Co.

*Transactions of the Medical Association of the State of Alabama.* Montgomery: Brown Printing Co.

*Transactions of the Medical Society of New Jersey.* Newark: L. J. Hardham.

*Transactions of the Medical Society of the State of New York.* Philadelphia: William J. Dornan.

*Transactions of the Medical Society of Virginia.* Richmond: J. W. Fergusson & Son.

*Transactions of the Michigan State Medical Society.* Grand Rapids: Seymour & Muir Printing Co.

*Transactions of the New Hampshire Medical Society.* Concord: Republican Press Association.

*Transactions of the New York Academy of Medicine.* New York: Stettiner, Lambert & Co.

*Transactions of the Ohio State Medical Society.* Toledo: The Blade Printing and Paper Co.

*Transactions of the Texas State Medical Association.* Galveston: Knapp Brothers.

### ASIA.

### CHINA.

*Quarterly.*

*The China Medical Missionary Journal.* Shanghai: The American Presbyterian Mission Press.

INDIA.

*Monthly.*

*The Indian Medico-Chirurgical Review.* Bombay: N. K. Rao & Co.

JAPAN.

*Monthly.*

*The Sei-I-Kwai Medical Journal.* Tokio: 6 Shin Sakana Chō, Kyōbashi Ku.

AUSTRALASIA.

AUSTRALIA.

*Monthly.*

*Intercolonial Medical Journal of Australasia.* Melbourne: Stillwell and Co.

*The Australasian Medical Gazette.* Sydney: 121 Bathurst Street.

NEW ZEALAND.

*Quarterly.*

*The New Zealand Medical Journal.* Dunedin: J. Wilkie and Co.

EUROPE.

AUSTRIA.

*Weekly.*

*Wiener klinische Wochenschrift.* Vienna: Wilhelm Braumüller.

BELGIUM.

*Twice a month.*

*Journal de Neurologie & d'Hypnologie.* Brussels: 3 Rue des Hirondelles.

*Monthly.*

*Bulletin de l'Académie royale de Médecine de Belgique.* Brussels: F. Hayez.

BRITISH ISLES.

*Weekly.*

*British Medical Journal.* London: 429 Strand.

*Medical Press and Circular.* London: A. A. Tindall.

*The Clinical Journal.* London: E. Knight.

*The Hospital.* London: 428 Strand.

*The Lancet.* London: 423 Strand.

*The Sanitary Record.* London: The Sanitary Publishing Company.

*Monthly.*

*Clinical Sketches.* London: Smith, Elder & Co.

*Edinburgh Medical Journal.* Edinburgh: Oliver and Boyd.

*The Birmingham Medical Review.* Birmingham: Hall & English.

*The British Journal of Dermatology.* London: H. K. Lewis.

*The Dublin Journal of Medical Science.* Dublin: Fannin & Co.

*The Journal of Laryngology, Rhinology, and Otology.* London: The Rebman Publishing Company, Limited.

*The Medical Chronicle.* Manchester: John Heywood.

*The Medical Magazine.* London: The "Medical Magazine" Co., Limited.

*Transactions of the Odontological Society of Great Britain.* London: John Bale & Sons.

*The Practitioner.* London: Cassell & Company, Limited.

*Quarterly.*

*Archives of Surgery.* London: West, Newman & Co.

*The Asclepiad.* London: Longmans, Green & Co.

*The British Gynæcological Journal.* London: John Bale & Sons.

*The Quarterly Medical Journal.* Sheffield: Pawson & Brailsford.

*The West London Medical Journal.* London: John Bale & Sons.

*Half-yearly.*

*The Liverpool Medico-Chirurgical Journal.* Liverpool: Medical Institution.

*The Retrospect of Medicine.* London: Simpkin & Co. Limited.

*Yearly.*

*Clinical Society's Transactions.* London: Longmans, Green, and Co.

*Guy's Hospital Reports.* London: J. & A. Churchill.

*King's College Hospital Reports.* London: Adlard & Son.

*Medico-Chirurgical Transactions.* London: Longmans, Green, and Co.

*Obstetrical Transactions.* London: Longmans, Green, and Co.

*Ophthalmological Transactions.* London: J. & A. Churchill.

*St. Bartholomew's Hospital Reports.* London: Smith, Elder, & Co.

*St. Thomas's Hospital Reports.* London: J. & A. Churchill.

*The Medical Annual.* Bristol: John Wright & Co.

*The Medical Society's Transactions.* London: Harrison & Sons.

*The Transactions of the Edinburgh Obstetrical Society.* Edinburgh: Oliver and Boyd.

*The Westminster Hospital Reports.* London: J. & A. Churchill.

*The Year-Book of Treatment.* London: Cassell & Company, Limited.

*Transactions of the Dermatological Society of Great Britain and Ireland.* London: H. K. Lewis.

*Transactions of the Epidemiological Society of London.* London: Shaw and Sons.

*Transactions of the Royal Academy of Medicine in Ireland.* Dublin: Fannin & Co.

*Year-Book of Scientific and Learned Societies.* London: C. Griffin & Co.

*Occasionally.*

*The International Directory of Second-hand Booksellers.* Rochdale: James Clegg.

DENMARK.

*Weekly.*

*Hospitalstidende.* Copenhagen: Jacob Lund.

### FRANCE.

*Three times a week.*

*Gazette des Hôpitaux.* Paris: 4 Rue de l'Odéon.

*Weekly.*

*Bulletin de l'Académie de Médecine.* Paris: G. Masson.

*Gazette hebdomadaire des Sciences médicales de Bordeaux.* Bordeaux: P. Cassignol.

*Gazette des Hôpitaux de Toulouse.* Toulouse: 3 Avenue Lafayette.

*La France médicale.* Paris: 9 Rue des Pyramides.

*Le Progrès médical.* Paris: 14 Rue des Carmes.

*L'Union médicale.* Paris: Octave Doin.

*Revue hebdomadaire de Laryngologie, d'Otologie et de Rhinologie.* Paris: Octave Doin.

*Fortnightly.*

*Le Nord médical.* Lille: 209 Rue Léon-Gambetta.

*Twice a month.*

*Gazette de Gynécologie.* Paris: 12 Rue de Châteaudun.

*La Revue médicale.* Paris: F. Sallé.

*Revue de Thérapeutique médico-chirurgicale.* Paris: 6 Rue Gît-le-Cœur.

*Revue internationale de Médecine et de Chirurgie.* Paris: 18 Rue de Provence.

*Monthly.*

*Archives cliniques de Bordeaux.* Bordeaux: 8 Rue de Cheverus.

*Archives de Neurologie.* Paris: 14 Rue des Carmes.

*Revue générale d'Ophtalmologie.* Paris: Masson et Cie.

*Revue des Instruments de Chirurgie.* Paris: 4 Rue Royer-Collard.

*Revue médico-chirurgicale des Maladies des Femmes.* Paris: 45 Boulevard Malesherbes.

*Revue obstétricale et gynécologique.* Paris: 35 Boulevard Haussmann.

*Quarterly.*

*Bulletins et Mémoires de la Société de Médecine et de Chirurgie pratiques de Paris.* Paris: 28 Rue Serpente.

*La Bibliographie scientifique.* Paris: 14 Boulevard Saint-Germain.

*Half-yearly.*

*Travaux d'Électrothérapie gynécologique.* Paris: Société d'Éditions scientifiques.

### GERMANY.

*Weekly.*

*Centralblatt für Chirurgie.* Leipzig: Breitkopf & Härtel.

*Centralblatt für Gynäkologie.* Leipzig: Breitkopf & Härtel.

*Centralblatt für innere Medicin.* Leipzig: Breitkopf & Härtel.

*Münchener medicinische Wochenschrift.* Munich: J. F. Lehmann.

*Monthly*

*Zeitschrift für Krankenpflege.* Berlin: H. Kornfeld.

### GREECE.

*Weekly.*

*Γαληνὸς.* Athens: N. G. Passare.

*Ἰατρικὴ Ἐπιθεώρησις.* Athens: 40 Rue Zinonos.

### HOLLAND.

*Weekly.*

*Nederlandsch Tijdschrift voor Geneeskunde.* Amsterdam: F. van Rossen.

### ITALY.

*Weekly.*

*Gazzetta Medica Lombarda.* Milan: 40 Via Senato.

*Twice a month.*

*Giornale Internazionale delle Scienze Mediche.* Naples: Detken & Rocholl.

*Monthly.*

*Annali di Ostetricia e Ginecologia.* Milan: Lodovico Felice Cogliati.

*Bi-monthly.*

*Archivio di Ortopedia.* Milan: 31 Via S. Calimero.

### NORWAY.

*Monthly.*

*Norsk Magazin for Lægevidenskaben.* Christiania: Det Steenske Bogtrykkeri.

### PORTUGAL.

*Weekly.*

*A Medicina Contemporanea.* Lisbon: José Antonio Rodrigues.

### ROUMANIA.

*Twice a month.*

*Spitalul.* Bucharest: Thoma Basilescu.

### RUSSIA.

*Weekly.*

*Medycyna.* Warsaw: 38 Rue de Varenna.
*St. Petersburger Medicinische Wochenschrift.* St. Petersburg: Carl Ricker.
*Vrach.* St. Petersburg: C. Ricker.

*Monthly.*

*Finska Läkaresällskapets Handlingar.* Helsingfors: Helsingfors Central-Tryckeri.

### SPAIN.

*Weekly.*

*El Siglo Médico.* Madrid: Magdalena, 36.

*Twice a month.*

*Revista de Ciencias Médicas de Barcelona.* Barcelona: 9 Calle Fortuny.

### SWEDEN.

*Quarterly.*

*Nordiskt Medicinskt Arkiv.* Stockholm: P. A. Norstedt & Söner.

### TURKEY.

*Twice a month.*

*Gazette médicale d'Orient.* Constantinople: A. Christidis.

### *PUBLICATIONS RECEIVED.*

From J. W. Arrowsmith, Bristol:
*A Few Medical & Surgical Reminiscences.* By Augustin Prichard. 1896.

From T. B. Browne, Ltd., London:
*The Advertiser's A B C.* 1896.

From Burroughs, Wellcome & Co., London:
*A B C Medical Diary.* 1896.
*A B C Chemist's Vest Pocket Diary.* 189
*Anti-Diphtheritic Treatment Chart.*
*The Nurses' Diary.* 1896.

From Cassell and Company, Limited, London:
*The Practitioner.* Vol. LV. 1895.

From The Church Defence Institution, London:
*The National Church.* January—March, 1896.

From J. & A. Churchill, London:
*The Physiology of the Carbohydrates.* An Epicriticism by F. W. Pavy, M.D., LL.D., F.R.S. 1895.
*History of the Cholera Controversy.* By Sir George Johnson, M.D., F.R.S. 1896.
*A Handbook of Leprosy.* By S. P. Impey, M.D., M.C. 1896.

From Church Missionary Society, London:
*Medical Mission Quarterly.* No. XIII. January, 1896.

From William F. Clay, Edinburgh:
*The Histopathology of the Diseases of the Skin.* By Dr. P. G. Unna. Translated by Norman Walker, M.D. 1896.

From W. H. & L. Collingridge City Press, London:
*The Prevention of Epidemics.* By Francis Vacher. 1895.

From The F. A. Davis Company, Philadelphia:
*Principles of Surgery.* By N. Senn, M.D., Ph.D., LL.D. Second Edition. 1895.
*Color-Vision and Color-Blindness.* By J. Ellis Jennings, M.D. 1896.

From Eduardo Dublan, Mexico:
*Boletin del Consejo Superior de Salubridad.* Octubre 31 de 1895.

From M. Henry, London:
*Food and Sanitation.* Dec. 14, 1895, Feb. 29, 1896.

From John Heywood, Manchester:
*Report on "Provident Medical Aid"* by the Council of the Medical Guild. 1895.

From Horne & Son, Whitby:
*Health Notes for the Seaside: with Special Reference to Whitby & District.* By A. C. Dutt, B.A., M.B. 1895.

From "Illustrated London News" Office, London:
*The English Illustrated Magazine.* February, March, 1896.

From Imprenta del Gobierno en el ex-Arzobispado, Mexico:
*Velada Fúnebre que en Honra del Eminente Sabio Francés Luis Pasteur celebró La Academia Nacional de Medecina de Mexico.* 1895.

From Jeyes' Sanitary Compounds Co., Ltd., London:
*Nosophen, Antinosine, and Endoxine, and their External and Internal Applications.* February, 1896.
*The English Physician on Lysidine in Gouty Disorders.* February, 1896.

From The Johns Hopkins Press, Baltimore:
*The Johns Hopkins Hospital Reports.* Volume V. 1895.

From H. K. Lewis, London:
*The Middlesex Hospital, W. Reports of the Medical, Surgical, and Pathological Registrars for the Year* 1894. 1895.
*The Prophylactic Clothing of the Body.* By W. F. Cleveland, M.D. 1896.
*Nursing Chart.*

From R. E. Lynch, Philadelphia:
*Report of the Kensington Hospital for Women*, Oct. 1894 to Oct. 1895.

From Alex. Macdougall, Glasgow:
*History of Chronic Nervous Diseases.* By Thomas Kirkpatrick Monro, M.A., M.D. 1895.

From Macmillan and Co., London:
*Prize Essays on Subjects connected with Leprosy.* No. 2. *Conditions under which Leprosy has Declined in Iceland.* By Edward H. Ehlers, M.D. No. 3. *Leprosy in South Africa.* By S. P. Impey, M.D., M.C. No. 4. *On Spontaneous Recovery from Leprosy.* By S. P. Impey, M.D., M.C. 1895.

From Dr. H. Macnaughton-Jones, London:
*Ménière's Disease.—Apoplectiform Labyrinthine Vertigo. Turbinal Hypertrophy in its Relation to Deafness, with a Special Bearing on the Operation of Turbinotomy. Etiological Considerations in the Treatment of "Nerve Deafness."* By H. Macnaughton-Jones, M.D., M.Ch. 1895. [Reprint.]

From Dr. THOMAS H. MANLEY, New York:
*Various Fractures.* By THOMAS H. MANLEY, M.D. 1894. [Reprint.]
*Deformities following Fractures.* By THOMAS H. MANLEY, M.D. 1895. [Reprint.]
*The Limitation of Surgical Operations as a Means of Relief or Cure in Epilepsy.* By THOMAS H. MANLEY, M.D. 1895. [Reprint.]

From Dr. CHARLES P. NOBLE, Philadelphia:
*Movable Kidney.* By CHARLES P. NOBLE, M.D. [N.D.] [Reprint.]
*Technique of Emptying the Uterus in Inevitable Abortion.* By CHARLES P. NOBLE, M.D. 1895. [Reprint.]

From OLIVER AND BOYD, Edinburgh:
*On the Diagnosis of Tubercular Joint Disease.* By A. G. MILLER. 1896.

From 11 PALACE CHAMBERS, London:
*The Parish Councillor.* Dec. 20, 1895.

From PARKE, DAVIS & Co., London:
*Price List.* 1896.
*Amylaceous Indigestion and its Treatment by Taka-Diastase.* By R. COWAN LEES, M.B. 1895. [Reprint.]
*Digestive Ferments. Pepsin, Pancreatin, Taka-Diastase.* 1895.
*Taka-Diastase.* 1895.
"*Salivary Indigestion*" *and its Causes. The Digestion of Starches. Taka-Diastase.* [N.D.]

From YOUNG J. PENTLAND, Edinburgh:
*Traumatic Infection.* By CHARLES BARRETT LOCKWOOD. 1896.
*Aseptic Surgery.* By CHARLES BARRETT LOCKWOOD. 1896.

From POPE MFG. Co., Hartford, Conn.:
*Columbia Calendar,* 1896.

From G. P. PUTNAM'S SONS, London:
*Lectures on Appendicitis. Notes.* By ROBERT T. MORRIS, A.M., M.D. 1895.

From THE REBMAN PUBLISHING Co., LTD., London:
*An American Text-Book of Surgery.* Edited by WILLIAM W. KEEN, M.D., LL.D., and J. WILLIAM WHITE, M.D., Ph.D. Second Edition. 1895.
*The American Year-Book of Medicine and Surgery.* Under the general editorial charge of GEORGE M. GOULD, M.D. 1896.

From THE REPUBLIC PRESS, New York:
*Eleventh Annual Report of the New York Post-Graduate Hospital.* 1895.

From JOHN MORGAN RICHARDS, London:
*Medical Reprints.* December, 1895—February, 1896.

From Dr. HUNTER ROBB, Cleveland, Ohio:
*Thoroughness in Medical Education.* By HUNTER ROBB, M.D. 1895. [Reprint.]

From GEO. ROBERTSON AND Co., Sydney:
*Hermes Medical Supplement.* Vol. I., No. 2. December 4th, 1895.

From THE ROXBURGHE PRESS, London:
*The Nursing News and Hospital Review.* January, February, 1896.

From THE ST. JOHN AMBULANCE ASSOCIATION, London:
*The Advantages of Ambulance Instruction, and the powers possessed by County Councils and Evening Continuation Schools to further the work of the St. John Ambulance Association, with special reference to the Mode of Help by County Councils, prefaced by some particulars of the Order of the Hospital of St. John of Jerusalem in England and its Departments.* 1895.

From THE SANITARY PUBLISHING COMPANY, LTD., London:
*The Natural Arsenical Waters of La Bourboule.* By A. M. BROWN, M.D. [N.D.]

From W. B. SAUNDERS, Philadelphia:
*Manual of the Practice of Medicine.* By GEORGE ROE LOCKWOOD, M.D. 1896.
*Manual of Medical Jurisprudence and Toxicology.* By HENRY C. CHAPMAN, M.D. Second Edition. 1896.
*Text-Book upon the Pathogenic Bacteria.* By JOSEPH MCFARLAND, M.D. 1896.

From GEORGE TIEMANN & Co., New York:
*Tiemann's Reprints.* Nos. 1—14.

From C. TINLING & Co., Liverpool:
*Dairy Milk.* By CHARLES HENRY LEET. [1896.]

From WARD, LOCK & BOWDEN, LIMITED, London:
*The Paper Knife.* Xmas, 1895.

From WILLIAMS & NORGATE, London:

*Teratologia.* December, 1895.

*Leitfaden der Hygiene.* Von Dr. AUG. GÄRTNER. Zweite Auflage. 1896.

*Grundriss der Pathologischen Anatomie.* Von Prof. Dr. R. LANGERHANS. Zweite Auflage. 1896.

*Geschichte der Volksseuchen.* Von Dr. B. M. LERSCH. 1896.

*L'Obstétrique.* No. 1. 15 Janvier, 1896.

From JOHN WRIGHT & Co., Bristol:

*Skin Pharmacopœia.* Edited by JAMES STARTIN. Fourth Edition. 1896.

From A. & M. ZIMMERMANN, London:
*The Therapist.* 15th January, 1896.

